# *In Vitro* Anti-Microbial Activity and Anti-Cancer Potential of Novel Synthesized Carbamothioyl-Furan-2-Carboxamide Derivatives

**DOI:** 10.3390/molecules28124583

**Published:** 2023-06-06

**Authors:** Muhammad Salman Javed, Muhammad Zubair, Komal Rizwan, Muhammad Jamil

**Affiliations:** 1 Department of Chemistry, Government College University Faisalabad, Faisalabad 38000, Pakistan; salmanjavid001@gmail.com; 2 Department of Chemistry, University of Sahiwal, Sahiwal 57000, Pakistan; 3 Department of Chemistry, Government Post Graduate College, Sahiwal 57000, Pakistan

**Keywords:** carbamothioyl, carboxamide, furan, anti-cancer, anti-microbial

## Abstract

A series of carbamothioyl-furan-2-carboxamide derivatives were synthesized using a one-pot strategy. Compounds were obtained in moderate to excellent yields (56–85%). Synthesized derivatives were evaluated for their anti-cancer (HepG2, Huh-7, and MCF-7 human cancer cell lines) and anti-microbial potential. Compound p-tolylcarbamothioyl)furan-2-carboxamide showed the highest anti-cancer activity at a concentration of 20 μg/mL against hepatocellular carcinoma, with a cell viability of 33.29%. All compounds showed significant anti-cancer activity against HepG2, Huh-7, and MCF-7, while indazole and 2,4-dinitrophenyl containing carboxamide derivatives were found to be less potent against all tested cell lines. Results were compared with the standard drug doxorubicin. Carboxamide derivatives possessing 2,4-dinitrophenyl showed significant inhibition against all bacterial and fungal strains with inhibition zones (I.Z) in the range of 9–17 and MICs were found to be 150.7–295 μg/mL. All carboxamide derivatives showed significant anti-fungal activity against all tested fungal strains. Gentamicin was used as the standard drug. The results showed that carbamothioyl-furan-2-carboxamide derivatives could be a potential source of anti-cancer and anti-microbial agents.

## 1. Introduction

**Figure 1:**
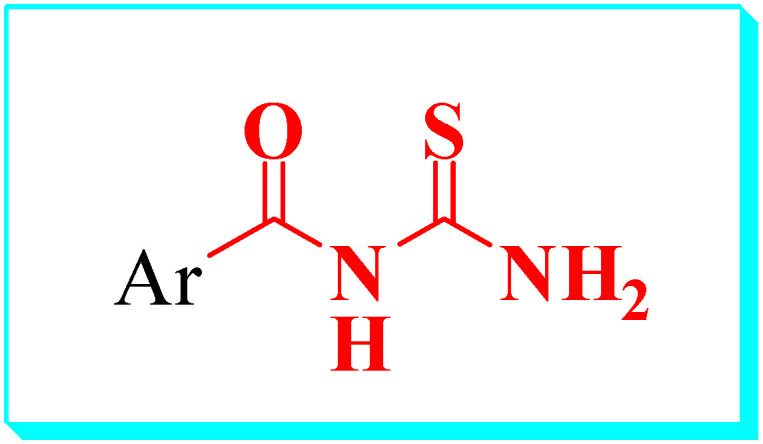
General chemical structure of carbamothioyl-aryl-2-carboxamide.

Carbamothioyl-aryl-2-carboxamide has promising pharmacological properties [1,2,3,4]. The general structure of carbamothioyl-aryl-2-carboxamide is shown in Figure 1.

**Figure 2:**
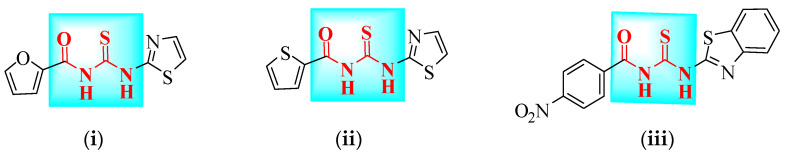
Active anti-fungal (**i**), anti-bacterial (**ii**) and anti-cancer (**iii**) derivatives of different carbamothioyl-aryl-2-carboxamides.

Carbamothioyl-aryl-2-carboxamide derivatives including furane, thiazole, benzothiozole, and thiophene moieties showed active anti-fungal (i), anti-bacterial (ii) and anti-cancer potential (iii) [5,6] (Figure 2). 

Scientists are attempting to incorporate furan-based building blocks into drug molecules to give them exceptional medicinal properties. The furan moiety is physiologically active, and it has the potential to modify protein-based molecules [7,8]. Polymeric organic materials have potential for application in the encapsulation of drugs for targeted drug delivery and also play a role as anti-microbial agents [9,10]. Currently, human hepato-cellular carcinoma is the fifth most common disease and third most common reason for mortalities globally [11,12]. Instead of significant progress in medical procedures and chemotherapy, most patients with hepatocellular carcinoma lose their lives within one year of diagnosis. More than 600,000 patients every year lose their lives due to liver malignant growths. Hepato-cellular carcinoma is primarily treated with chemotherapy and through surgical procedures [13,14]. Doxorubicin is the most commonly used drug against hepatic syndromes, either alone or in conjunction with other medications such as cisplatin. However, the results of current standard chemotherapeutic medication are not matched with therapeutic needs due to their severe toxic effect on ordinary hepatocytes [15]. Despite a lot of research on the treatment of liver carcinoma, there remains an opportunity to improve or synthesize novel drugs for chemotherapeutic treatment of liver cancer with the minor side effects. Heteroatom-containing compounds are biologically active, and they are building blocks for various drug molecules [16,17]. Due to the presence of heteroatoms (N, S and O) in carbamothioyl-aryl-2-carboxamide, its derivatives have a variety of biological functions and are broadly used in the field of medicinal chemistry [5,6]. (Figure 2). Functional moieties such as carbonyl, thiocarbonyl, and amines are building blocks of many drugs. These groups frequently perform a structural role, linking other functionalities and aiding in the optimal orientation for interaction with the drug’s target [18,19]. The chemical reactivity of carbonyl functional groups can also allow these groups to come into close contact with the target, generating hydrogen bonds and other intermolecular interactions. Considering the worldwide spread of cancer and microbe-related diseases where microbes have become drug resistant, there is a need to find more potent anti-cancer and anti-microbial agents. The compounds containing carboxamide and carbamothioyl moieties have been proven to be potent anti-cancer and anti-microbial agents [5,6,20]. In this context, the current study describes the synthesis and anti-microbial assessment of a few furan-based acyl thiourea derivatives (containing both carboxamide and carbamothioyl moieties). So far, our research group has reported the synthesis and pharmacological potential of symmetrical di-substituted thiourea, and these compounds were found to be potently active as anti-urease and anti-microbial agents [20]. In this research, we have synthesized next-generation carbamothioyl-furan-2-carboxiamide derivatives (**4a–f**), and these derivatives were screened for their anti-cancer and anti-microbial activities.

## 2. Results and Discussion

### 2.1. Synthesis

**Figure 3:**
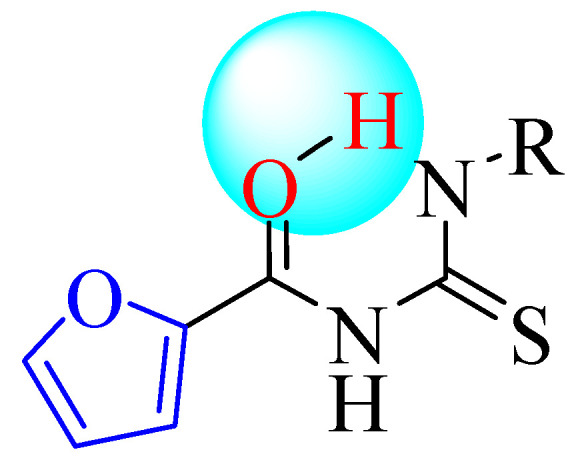
Intra-molecular hydrogen bonding in synthesized carbamothioyl-furan-2-carboxamide derivatives.

**Scheme 1:**
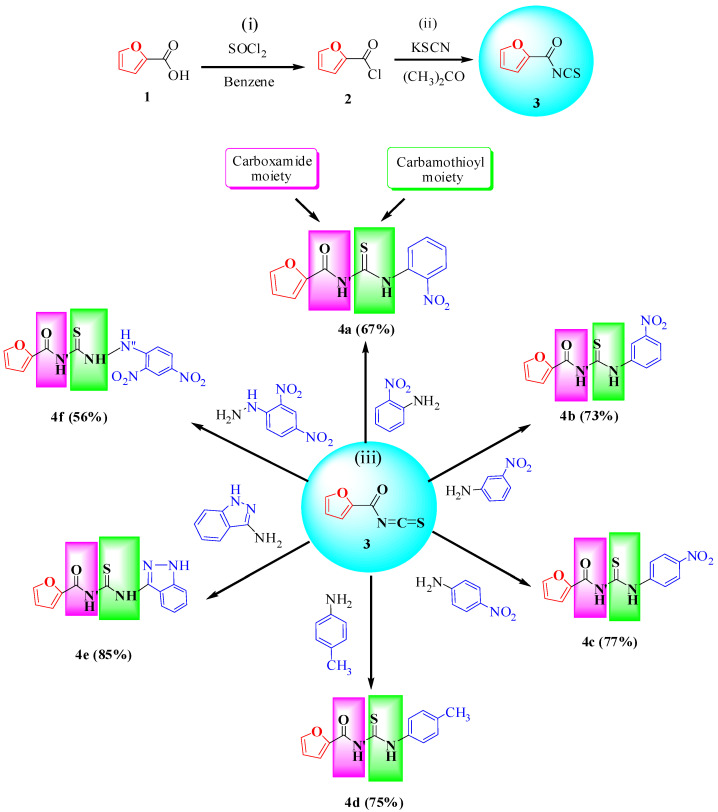
Synthesis of carbamothioyl-furan-2-carboxamide derivatives (**4a–f**). Reaction conditions: (i) **1** (1 g, 9.0 mmol), SOCl_2_ (0.65 mL, 9.0 mmol), benzene (20 mL) refluxed in water bath for 10–12 h (ii) **2** (0.3 mL, 3.8 mmol), acetone (20 mL), KSCN (0.36 g, 3.8 mmol) stirred for 1 h (iii) **3** (in situ), H_2_NR (3.8 mmol), acetone (10 mL), reflux for 6 h.

We prepared the solution of 2-furoic acid in dry benzene and mixed it with thionyl chloride solution. The mixture was boiled for 10–12 h in a water bath (Scheme 1). Then, the excess solvent and SOCl_2_ were removed under reduced pressure, a further reaction mixture was concentrated in the vacuum, and furan-2-carbonyl chloride in a colorless liquid was obtained (**2**). Furan-2-carbonyl chloride (**2**) was treated with anhydrous KSCN in the presence of dry acetone to synthesize furoyl iso-thiocyanate (**3**) in a quantitative yield. Iso-thiocyanate is a useful precursor in synthetic chemistry for the synthesis of different substituted carbamothioyl-furan-2-carboxamides [21]. The corresponding carbamothioyl-furan-2-carboxamide derivatives (**4a**–**f**) were produced in moderate to excellent yields using iso-thiocyanate (**3**), which was not separated and used directly to react with amines. The addition to the -NCS system and the nucleophilic substitution of carbonyl carbon may compete with one another. It has been observed that iso-thiocyanate (**3**) undergoes an additional reaction with primary amines to synthesize the corresponding carbamothioyl-furan-2-carboxamide derivatives (**4a**–**f**). The target compounds (**4a**–**f**) were obtained in moderate to excellent yields (56–85%) (Figure 3). Structure–activity relationships (SAR) in relation to the substituent on the phenyl ring in the amine substrate can be used to explain the percentage yield. In comparison to meta- (**4b**; 73%) and ortho- (**4a**; 67%) substituted products, para-substituted products (**4c**; 77%) and (**4d**; 75%) were obtained in good yields due to the steric effect. The indazole moiety was also successfully incorporated under optimized conditions and product **4e** was obtained in high yield (85%). In the IR spectrum, an N-H absorption band was observed from 3189–3389 cm^−1^, whereas appearance of carbonyl peaks in the range of 1664 to 1678 cm^−1^ is explained on the basis of intra-molecular hydrogen bonding between the oxygen of the carbonyl group and the hydrogen of the thioamide group (Figure 3). The C=S peak appeared from 1205 to 1266 cm^−1^. The N-H protons in 1H NMR spectra appeared from 13.21 to 10.46 ppm as singlet, while N′-H protons appeared in the range of 9.37–7.62 ppm. Carbonyl and thio-carbonyl groups produce a strong de-shielding effect, due to which the proton of N-H appears at a high frequency. The aromatic protons appeared in the region of 9.09 to 6.63 ppm. The C=S and C=O groups provided us with the most de-shielded ^13^C NMR signals. The carbon of thio-carbonyl group showed signals at δ 180.0 to 176.1 ppm due to low excitation energy [22]. The signals of carbonyl carbon appear in the region of δ 156.1 to 158.2 due to the involvement of oxygen atoms from the carbonyl group in intra-molecular hydrogen bonding (Figure 3).

### 2.2. Pharmacology

#### 2.2.1. Anti-Cancer Activity of Synthesized Carbamothioyl-Furan-2-Carboxamide Derivatives against HepG2, Huh-7, and MCF-7 Cancer Cell Line

**Figure 4:**
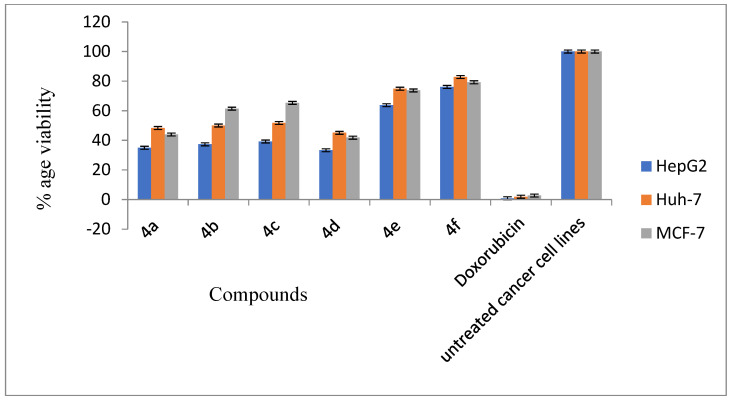
Anti-cancer activity of carbamothioyl-furan-2-carboxamide derivatives (**4a**–**f**) against HepG2, Huh-7, and MCF-7 cancer cell lines.

Human cancer cells (HepG2, Huh-7, and MCF-7) were employed to test the anti-cancer potential of synthesized molecules. Untreated cancer cell linings (HepG2, Huh-7, and MCF-7) were used as the negative control and doxorubicin was employed as the positive control (Table 1). The compound **4d** showed the highest anti-cancer activity with less cell viabilities (33.29, 45.09, and 41.81%, respectively) among all synthesized compounds (**4a–f**)**.** Compounds **4a**, **4b**, and **4c** also showed significant anti-cancer activity in the case of HepG2, with a cell viability of 35.01%, 37.31%, and 39.22%, respectively, while **4e** and **4f** were found to be less potent against all tested cancer cell lines with relatively high cell viability (63.75–82.81%). The relative activity of **4a**–**f** against the cancer cell line is as follows: **4d** < **4a** < **4b** < **4c** < **4e** < **4f** (Table 1, Figure 4). Studies revealed that the drug doxorubicin, which is commonly used for the treatment of HepG2 cancer, showed a cell viability of 0.62% (48 h) [23]. The anti-cancer activity of structure–activity relationships (SAR) pertaining to the substituent connected to the phenyl ring can be used to explain the anti-cancer activity of carbamothioyl-furan-2-carboxiamde derivatives. The detailed examination of the synthesized compounds’ structure and activity revealed that the presence of electron-donor substituents increases their anti-cancer action [24,25,26]; para-methyl-substituted **4d** exhibited greater anti-cancer activity (% cell viability = 33.29, 45.09, and 41.81%) than other compounds. The presence of the nitro group at different positions (*o*, *m*, *p*) in the phenyl ring exhibited promising anti-cancer potential; *o*-nitro-substituted **4a** showed significant anti-cancer potential (cell viability = 35.01, 48.32, 43.96 %) and was found to be the most active anti-cancer agent among meta and para nitro-substituted compounds **4b** and **4c,** with cell viability in the range of 37.31–65.33 %. The decreasing activity order of nitro-substituted compounds are ortho > meta > para. Similarly, in compounds **4e** and **4f,** the presence of a bulky group in the ring may be responsible for increasing the cell viability of compounds towards the cancer cell line.

#### 2.2.2. Hemolytic Activity

The percentage of hemolysis was calculated for synthesized carbamothioyl-furan-2-carboxamide derivatives (**4a**–**f**), and results are shown in Table 1. Low toxicity of synthetic compounds was seen with RBCs. Compound **4a** (2.60%), which demonstrated less affinity with the RBC cell membrane than conventional ABTS (95.9% hemolysis), was found to be the least hazardous. Compound **4e** (with hemolysis) was determined to be the most toxic derivative, followed by **4b** (4.12%), **4c** (4.84%), **4d** (4.56%), and **4f** (7.25%), which showed low hemolytic activity. 

#### 2.2.3. Anti-Microbial Activity

Microbes cause major health risks, so it is necessary to find new sources to inhibit the growth of microbes. Previously, various synthetic drugs have been reported as potent sources of anti-microbial agents [27,28,29]. In this research, synthesized compounds (**4a**–**f**) were tested against three bacterial (*S. aureus*, *E. coli*, and *B. cereus*) and three fungal strains (*F. bracchygibossum*, *A. niger*, and *A. flavus*) by using the well diffusion method (Figures S1–S4: see Supplementary Data), and anti-microbial potential was determined by measuring the growth inhibition zone and MIC values. The standard drug was gentamicin. Compound **4f** showed activity against all three bacterial strains, with an inhibition zone in the range of 9–16 mm at 10 mg/mL and MIC values were found in range of 230–295 µg/mL, while compounds **4a**, **4b,** and **4c** were found active against two bacterial strains (I.Z. = 10–15 mm and MIC = 240–280 µg/mL). Compound **4d** showed an inhibition zone only against *S. aureus* (I.Z. = 12 mm and MIC = 270 µg/mL), while **4e** showed an inhibition zone only against *E. coli* (I.Z. =12 mm and MIC = 300 µg/mL). Due to the presence of a thicker peptidoglycan layer in gram-positive bacteria, they are more resistant towards the synthesized compounds than gram-negative bacteria which have a thinner peptidoglycan layer [30,31], although the synthesized derivatives could still penetrate resulting in inhibition zones. Our synthesized carbamothioyl-furan-2-carboxamide derivatives (**4a**–**f**) showed significant anti-microbial activity in comparison to already-reported carbamothioyl-alkyl-carboxamide derivatives [32]. This high anti-bacterial activity may be due to the presence of aromatic moiety in our synthesized compounds, and this behavior can also be explained by the lipophilicity. Compounds with aromatic moiety are more lipophilic compared with non-aromatic moieties [33]. In the case of anti-fungal activity, the activity potential of synthesized compounds is more prominent than anti-bacterial activities. Compounds **4a**, **4b**, **4c**, and **4f** showed prominent activity against all fungal strains, with an inhibition zone range from 12–19 mm at 10 mg/mL. MIC values were found in the range of 120.7–190 µg/mL, while compounds **4d** and **4e** showed potent activity against only two fungal strains (I.Z. = 11–18 mm and MIC = 122.1–186 µg/mL). All the values of inhibition zones and MICs are summarized in Table 2 and Table 3, respectively. The nucleophilic character of nitrogen and sulfur in synthesized derivatives and polarity in the molecules may have assisted with the penetration through the bacterial cell wall, easily inhibiting the growth of bacterial cells [34]. Thiourea derivatives are an inhibitor of bacterial actin MreB and seem to inhibit bacterial growth in a non-specific fashion [35]. It was concluded that synthetic carbamothioyl-furan-2-carboxamide derivatives could be potent anti-microbial agents.

## 3. Materials and Methods

Analytical-grade solution and reagents were used. Pre-coated silica gel on aluminum plates was used for thin layer chromatography (TLC) and spots were observed under UV light at 254 nm. I.R. spectra have been recorded on a Bruker FT-IR spectrophotometer (Billerica, MA, USA). The ^1^H and ^13^C NMR signals have been recorded on a Bruker D.P.X. 400 spectrometer (Billerica, MA, USA) and CDCl_3_ was used as solvent. Furan-2-carboxlic acid, potassium thiocyanate (KSCN), thionyl chloride (SOCl_2_), acetone, benzene, ethyl acetate, and amines were purchased from Sigma-Aldrich (St. Louis MO, USA), Merck (Boston, MA, USA), and Alfa-Aesar (Karlsruhe, Germany) through local suppliers. 

### 3.1. General Method of Obtaining Caramothioyl-Furan-2-Carboxamide Derivatives ***4a**–**f***

A solution of 2-furoic acid (**1**) (1 g, 9.0 mmol) in dried benzene (20 mL) was prepared in a round bottom flask; then, a solution of thionyl chloride (SOCl_2_) (0.65 mL, 9.0 mmol) in benzene (10 mL) was mixed with (**1**). The mixture was then heated in a water bath for 10 to 12 h and excess solvent and SOCl_2_ was removed under reduced pressure. The mixture was concentrated in a vacuum and furan-2-carbonyl chloride (**2**) was obtained as a colorless liquid. This colorless liquid without purification was used for further reactions. The solution of dried potassium thiocyanate (3.8 mmol) in acetone (20 mL) was added to liquid **2** and stirred for 1 h at 25 °C to get iso-thiocyanate (**3**). The solution was then filtered to remove potassium chloride precipitates. This filtrate was mixed with the solution of primary amine (3.8 mmol) and refluxed for 6 h to obtain a moderate to good yield of desired carbamothioyl-furan-2-carboxamide derivatives (**4a**–**f**). Then, the mixture was poured into ice-cold de-ionized water. Precipitates appeared immediately which were allowed to settle down and were then filtered and dried in a vacuum desiccator. Products were further purified by recrystallization using ethyl acetate. Synthesized molecules were characterized by IR and NMR spectroscopy (spectra are presented in Supplementary Information). The synthesized molecules were stored in vials and vials were further sealed with parafilm to avoid moisture contamination. Vials were stored at the 4 °C in refrigerator until further usage

### 3.2. Characterization Data

(**4a**)**:** *N*-(2-nitrophenylcarbamothioyl)furan-2-carboxamide, (Yield = 0.74 g; 67%)**,** greenish yellow solid, m.p.: 148 °C; IR (KBr, υ_max_, cm^−1^); 3220 (N-H), 1673 (C=O), 1593 (C=C), 1530 (NO), 1338 (C-N), 1250 (C=S), 1H NMR (400 MHz, CDCl_3_): δ (ppm): 13.21 (s, 1H, NH), 9.37 (s, 1H, N’H), 8.49 (d, *J* = 8 Hz, 1H), 8.13 (d, *J* = 4 Hz, 1H), 7.72–7.66 (m, 2H), 7.48 (d, *J* = 4 Hz, 1H), 7.44 (t, *J* = 6 Hz, 1H), 6.67 (q, *J* = 1.2 Hz, 1H), ^13^C-NMR (100 MHz, CDCl_3_) δ: 179.1 (C=S), 156.1 (C=O), 146.6, 144.8, 142.4, 133.5, 132.4, 128.1, 126.9, 125.2, 119.6, 113.6. LC/MS m/z; [M − H] = 290.0. Anal. Calcd. (%) for C_12_H_9_N_3_O_4_S: C, 49.48; H, 3.11C; Found: C, 49.45; H, 3.14. EI-HR-MS C_12_H_9_N_3_O_4_S [M]•+ calcd = 291.0314; found = 291.0297.

(**4b**)**:** *N*-(3-nitrophenylcarbamothioyl)furan-2-carboxamide, (Yield = 0.80 g; 73%)**,** yellowish white solid, m.p: 201 °C; IR (KBr, υ_max_, cm^−1^); 3295 (N-H), 1664 (C=O), 1588 (C=C), 1442 (NO), 1343 (C-N), 1266 (C=S), 1H NMR (400 MHz, CDCl_3_): δ (ppm): 12.62 (s, 1H, NH), 9.30 (s, 1H, N’H), 8.76 (t, *J* = 1.6 1H), 8.16 (d, *J* = 4, 1H), 8.08 (d, *J* = 4, 1H), 7.69–7.60 (m, 2H), 7.47–7.44 (m, 1H), 6.70 (q, *J* = 1.2, 1H), ^13^C-NMR (100 MHz, CDCl_3_) δ: 178.5 (C=S), 156.9 (C=O), 148.4, 146.2, 144.6, 138.8, 129.8, 129.7, 121.4, 119.6, 118.9, 113.6. LC/MS m/z; [M − H] = 290.08. Anal. calcd. (%) for C_12_H_9_N_3_O_4_S: C, 49.48; H, 3.11; Found: C, 49.45; H, 3.14. EI-HR-MS C_12_H_9_N_3_O_4_S [M]•+ calcd = 291.0314; found = 291.0326.

(**4c**)**:** *N*-(4-nitrophenylcarbamothioyl)furan-2-carboxamide, (Yield = 0.85 g; 77%), yellowish white solid, m.p.: 212 °C; IR (KBr, υ_max_, cm^−1^); 3321 (N-H), 1664 (C=O), 1568 (C=C), 1504 (NO), 1346 (C-N), 1210 (C=S), 1H NMR (400 MHz, CDCl_3_): δ (ppm): 12.82 (s, 1H, NH), 9.32 (s, 1H, N’H), 8.30 (d, *J* = 8, 2H), 8.05 (d, *J* = 4, 2H), 7.68–7.63 (m, 1H), 7.46 (d, *J* = 8, 1H), 6.69 (q, *J* = 1.2, 1H), ^13^C-NMR (100 MHz, CDCl_3_) δ: 180.0 (C=S), 157.9 (C=O), 146.9, 145.2, 130.9, 128.8, 124.6, 123.2, 119.7, 113.6. LC/MS m/z; [M − H] = 290.08. Anal. calcd. (%) for C_12_H_9_N_3_O_4_S: C, 49.48; H, 3.11; Found: C, 49.45; H, 3.14. EI-HR-MS C_12_H_9_N_3_O_4_S [M]•+ calcd = 291.0314; found = 291.0297.

(**4d**)**:** *N*-(p-tolylcarbamothioyl)furan-2-carboxamide, (Yield = 0.74 g; 75%)**,** off white solid, m.p. 136 °C; IR (KBr, υ_max_, cm^−1^): 3389 (N-H), 1667 (C=O), 1581 (C=C), 1329 (C-N), 1251 (C=S), 1H NMR (400 MHz, CDCl_3_): δ (ppm): 10.46 (s, 1H, NH), 8.76 (d, *J* = 0.8 1H), 7.62 (s, 1H, N’H), 7.45 (d, *J* = 4, 2H), 7.41 (d, *J* = 4, 1H), 7.17 (d, *J* = 8, 2H), 6.63 (q, *J* = 1.4, 1H), 2.35 (s, 3H), ^13^C-NMR (100 MHz, CDCl_3_) δ: 178.1 (C=S), 158.1 (C=O), 146.2, 145.6, 134.5, 134.2, 129.6, 120.5, 118.1, 113.1, 20.9. LC/MS m/z; [M − H] = 259.08. Anal. calcd. (%) for C_13_H_12_N_2_O_2_S: C, 59.98; H, 4.65; Found: C, 59.93; H, 4.61. EI-HR-MS C_13_H_12_N_2_O_2_S [M]•+ calcd = 260.0619; found = 291.0624.

(**4e**)**:** *N*-(1H-indazol-3-ylcarbamothioyl)furan-2-carboxamide, (Yield = 0.85 g; 85%), off white solid, m.p. 210 °C; IR (KBr, υ_max_, cm^−1^): 3189 (NH), 1678 (C=O), 1577 (C=C), 1338 (C-N), 1205 (C=S), 1095 (N-N), 1H NMR (400 MHz, CDCl_3_): δ (ppm): 12.98 (s, 1H, NH indazol), 12.53 (s, 1H, NH) 9.56 (s, 1H, N’H), 7.82 (d, *J* = 8.0, 1H), 7.69–7.62 (m, 2H-), 7.53–7.47 (m, 3H), 6.65 (d, *J* = 0.4 1H), ^13^C-NMR (100 MHz, CDCl_3_) δ: 176.1 C=S, 157.4 C=O, 153.6, 146.5, 143.3, 140.5, 127.3, 119.2, 118.0, 116.4, 114.0, 113.1, 110.6. LC/MS m/z; [M − H] = 285.08. Anal. calcd. (%) for C_13_H_10_N_4_O_2_S: C, 54.54; H, 3.52; Found: C, 54.52; H, 3.49. EI-HR-MS C_13_H_10_N_4_O_2_S [M]•+ calcd = 286.0524; found = 286.0531.

(**4f**)**:** *N*-(2-(2,4-dinitrophenyl)hydrazinecarbamothioyl)furan-2-carboxamide, (Yield = 0.73 g; 56%), yellow solid, m.p. 124 °C; IR (KBr, υ_max_, cm^−1^); 3214 (N-H), 1669 (C=O), 1573 (C=C), 1530 (NO), 1336 (C-N), 1259 (C=S), 1094 (N-N), 1H NMR (400 MHz, CDCl_3_): δ (ppm):10.99 (s, 1H, N’H), 9.09 (d, *J* = 1.2, 1H-Ar), 8.25 (dd, *J* = 2.4, *J* = 9.2, 1H-Ar), 8.14 (d, *J* = 8.0, 1H-Ar), 7.92 (d, *J* = 8.2, 2H-Ar), 7.67–7.58 (m, 1H-Ar), 7.47–7.36 (m, 1H, -Ar), 4.62–3.86 (m, 1H, N”H), 2.65 (s, 1H, NH), ^13^C-NMR (100 MHz, CDCl_3_) δ: 180.0 (C=S), 158.2 (C=O), 153.1, 146.3, 144.6, 137.2, 133.6, 129.3, 122.4, 119.1, 119.5, 113.2. LC/MS m/z; [M − H] = 350.08. Anal. calcd. (%) for C_12_H_9_N_5_O_6_S: C, 41.03; H, 2.58; Found: C, 41.05; H, 2.55. EI-HR-MS C_12_H_9_N_5_O_6_S [M]•+ calcd = 351.0413; found = 351.0419.

### 3.3. Biological Activities

#### 3.3.1. Anti-Cancer Activity

##### Cell Culture and Treatment

The cancer cell lining was grown in Dulbecco’s Modified Eagle medium which provided fetal bovine serum (10%) and gentamicin (100 µg/mL) and was maintained at normal human body temperature (37 °C) with CO_2_ (5%). Cancer cells were treated with carbamothioyl-furan-2-carboxamide derivatives dissolved in solvent (DMSO) with a final concentration of 0.05%

##### Determination of Cell Viability

Cell viability was calculated by MTT assay [36]. Cancer cells were treated with carbamothioyl-furan-2-carboxamide derivatives at a 20 mg/mL concentration. A total amount of 0.5 µL was collected and diluted in 100 µL of cells, so the final concentration was 100 µg/mL which was incubated for 48 h. A further 10 µL of MTT reagent and cells have been incubated for 4 h at 37 °C, following which 150µL solvent (DMSO) was added to dissolve synthetic crystals. Microplate reader absorbance has been measured at 490 nm and the percentage of cell viability was measured.

#### 3.3.2. Anti-Microbial Activity by Well Diffusion strategy

Anti-microbial activity of synthesized carbamothioyl-furan-2-carboxamide derivatives was checked using the well diffusion method against 2 bacterial strains, *Staphylococcus aureus* (gram positive) and *Escherichia coli* (gram negative) and 2 fungal strains (*Fusarium bracchygibossum* and *Aspergillus niger*). Pure microbes (bacteria and fungi) were collected from the zoology department at the Government College University Faisalabad, Pakistan. Synthesized derivatives were dissolved in 10 mg/mL of DMSO to prepare the stock solution [37]. In a Petri dish and with the help of sterile a Q-tip, 0.5 McFarland standard population of microbial strains was consistently spread on the surface of the agar. Synthetic compound wells were then prepared in agar with an 8 mm plug drill. Approximately 10 mg/mL of stock solution was added to each well. Gentamicin (25 μg/mL), the positive control, was included in wells at the center of the Petri dish to compare the anti-microbial activity [38]. The drug conveyance plates were then fixed with parafilm and left for 2 h to allow for the appropriate diffusion of compounds. Then, these plates were placed in an incubator at 37 °C and 1 atm of pressure for 24 h. Incubation plates were observed after 24 h and the inhibition zone diameters were measured with an anti-microbial zone measuring scale.

##### Determination of MIC Values

A modified resazurin microtiter plate test was used to determine the minimum inhibitory concentration (MIC) of carbamothioyl-furan-2-carboxamide derivatives (**4a**–**f**) [39]. Every compounds solution (100 µL) prepared in 10% DMSO (*v*/*v*) was placed in first row of 96-well plates. Then, 50 µL each of Muller-Hinton-broth for the bacteria and thefungus and nutrition broth were added to all other wells. The test substance was serially diluted twice so that each well contained 50 µL at progressively lower concentrations.

Indicator resazurin solution was formed by dissolving 270 mg resazurin tablet into 40 ml of water and 10 µL indicator solution was added in each well. Then 10 µL of bacterial or fungal suspensions were incorporated into the each well. 96-well plates have been covered with aluminum foil and had a set of controls: a column with respective solvents as the negative control, a column with broad spectrum antibiotics as positive control, a column with all solutions with the exception of the test samples, a column with all solutions without the bacterial/fungal solution (with 10 µL of broth instead). Plates have been formed in triplicate and further incubation was carried out at 37 °C for 20 to 24 h and 28 °C for 40 to 48 h for bacteria and fungi respectively. Growth have been showed by colour changes from purple-pink or may be colorless. The minimum concentration at which the color change appeared was considered as the MIC value. 

#### 3.3.3. Hemolytic Assay

To determine their hemolytic potential, all of the synthetic compounds were tested in accordance with the literature [40]. Bovine blood samples (3 mL) were taken and centrifuged in EDTA at 1000× *g* for 10 min; following erythrocyte isolation, they were rinsed 3 times with a 5 mL cold-sterilized PBS solution with a pH of 7.4. A total of 20 µL of sample solution (400 µg/mL) in DMSO was combined with the blood suspension and incubated at 37 °C for 30 min. As positive and negative controls, respectively, ABTS and DMSO were employed. At 576 nm, the sample’s absorbance was noticed, and the percentage of hemolysis was computed.

$$\begin{array}{c} \%\mathrm{age}\mathrm{of}\mathrm{hemolysis}=[(\mathrm{absorbance}\mathrm{of}\mathrm{test}\mathrm{compound}(\mathrm{sample})-\mathrm{absorbance}\mathrm{of}\\ \mathrm{DMSO})/(\mathrm{absorbance}\mathrm{of}\mathrm{ABTS})]\times 100 \end{array}$$

## 4. Conclusions

We designed and synthetized a series of carbamothioyl-furan-2-carboxamide derivatives and evaluated their anti-cancer and anti-microbial activities. Synthesized compounds showed considerable anti-cancer activity against tested cell lines but **4a**, **4b**, **4c**, and **4d** showed significant anti-cancer potential, and results were compared with standard doxorubicin. Compounds **4b**, **4a**, and **4f** were found to be more potent (I.Z = 10.5, 13, 16 mm and MIC = 280, 265 and 230 μg/mL) against *E.coli*, *S. aureus*, and *B. cereus*, respectively. The anti-fungal activity of these compounds against all fungal strains is highly appreciable compared to standard doxorubicin. The results of biological activity tests reveal that these carbamothioyl-furan-2-carboxamide derivatives could be a good starting point for drug-discovery-related research. The modification of these molecules could introduce lead molecules for therapeutics in the future.

## Figures and Tables

**Table 1:** Anti-cancer activity of carbamothioyl-furan-2-carboxamide derivatives (**4a**–**f**) at 100 µg/mL concentration against human HepG2, Huh 7, and MCF 7 cancer cell lines and hemolytic activity.

Sr #	Entries (µM)	%Age Cell Viability (HepG2)	%Age Cell Viability (Huh-7)	%Age Cell Viability (MCF-7)	%Age Hemolysis
1	**4a** (340)	35.01 ± 1.55	48.32 ± 0.38	43.96 ± 1.42	2.60 ± 0.18
2	**4b** (340)	37.31 ± 1.72	50.02 ± 1.54	61.42 ± 0.87	4.12 ± 0.22
3	**4c** (340)	39.22 ± 4.04	51.76 ± 1.39	65.33 ± 0.17	4.84 ± 0.15
4	**4d** (380)	33.29 ± 5.22	45.09 ± 0.23	41.81 ± 1.42	4.56 ± 0.34
5	**4e** (350)	63.75 ± 0.30	74.91 ± 0.35	73.74 ± 0.41	8.32 ± 0.17
6	**4f** (280)	76.07 ± 0.88	82.81 ± 1.61	79.26 ± 0.76	7.25 ± 0.29
7	Doxorubicin (180)	00.86 ± 0.56	01.89 ± 0.73	02.71 ± 0.21	-
8	Untreated cancer cell linings	100.0 ± 0.00	100.0 ± 0.00	100.0 ± 0.00	-
9	DMSO	-	-	-	0.01 ± 0.08
10	ABTS	-	-	-	95.9 ± 0.02

For hemolysis activity, compounds were tested at 400 µg/mL. Values are ± S.D of triplicate experiments.

**Table 2:** Anti-microbial activity of the carbamothioyl-furan-2-carboxamide derivatives (**4a**–**f**) by well diffusion method.

Sr.	Entries Dose (10 mg/mL)	*E. coli* I.Z. (mm)	*S. aureus* I.Z. (mm)	*B. cereus* I.Z. (mm)	*F. bracchygibossum* I.Z. (mm)	*A. niger* I.Z. (mm)	*A. flavus* I.Z. (mm)
1	**4a**	-	13.00 ± 0.11	15.00 ± 0.13	16.00 ± 0.17	16.00 ± 0.13	14.00 ± 0.11
2	**4b**	10.50 ± 0.10	-	14.00 ± 0.15	16.00 ± 0.13	16.00 ± 0.12	14.00 ± 0.13
3	**4c**	10.00 ± 0.10	-	15.00 ± 0.16	17.00 ± 0.11	19.00 ± 0.14	15.00 ± 0.17
4	**4d**	-	12.00 ± 0.12	-	11.00 ± 0.12	16.00 ± 0.13	-
5	**4e**	09.00 ± 0.09	-	-	18.00 ± 0.11	-	12.00 ± 0.12
6	**4f**	09.00 ± 0.11	10.00 ± 0.11	16.00 ± 0.13	13.00 ± 0.12	17.00 ± 0.12	12.00 ± 0.11
7	Gentamicine (control)	21.00 ± 0.11	21.00 ± 0.21	20.00 ± 0.18	14.00 ± 0.11	14.00 ± 0.14	16.00 ± 0.11

Values are ± S.D of triplicate experiments.

**Table 3:** MIC values of the carbamothioyl-furan-2-carboxamide derivatives (**4a**–**4f**).

Sr.	MIC (µg/mL) Entries	*E. coli*	*S. aureus*	*B. cereus*	*F. bracchygibossum*	*A. niger*	*A. flavus*
1	**4a**	-	265	240	150.2	145.2	155
2	**4b**	280	-	250	150.0	145.0	155
3	**4c**	280	-	240	137.5	120.7	140
4	**4d**	-	277	-	270.1	154.0	-
5	**4e**	300	-	-	122.1	-	186
6	**4f**	295	280	230	150.7	157.4	190
7	Gentamicine (control)	120.35	122.50	120.47	160.25	163.31	164.30

## Data Availability

Not applicable.

## Supplementary Materials

The following supporting information can be downloaded at: https://www.mdpi.com/article/10.3390/molecules28124583/s1, Figure S1: Antibacterial activity of the synthesized compounds (**4a**–**4f**) by well diffusion method; Figure S2: Antifungal activity of the synthesized compounds (**4a**–**4f**) by well diffusion method; Figure S3: Antibacterial activity of the synthesized compounds (**4a**–**4f**) by well diffusion method; Figure S4: Antifungal activity of the synthesized compounds (**4a**–**4h**) by well diffusion method.
