# Supplementary material for: In Vitro Anti-Microbial Activity and Anti-Cancer Potential of Novel Synthesized Carbamothioyl-Furan-2-Carboxamide Derivatives
Source: Molecules. 2023 Jun 6;28(12):4583. doi: 10.3390/molecules28124583 (PMC10300966; doi:10.3390/molecules28124583)
Supplement: Supplementary file 1 [file molecules-28-04583-s001.zip › molecules-2082560-supplementary.pdf]

In-vitro antimicrobial activity and anticancer potential of novel synthesized carbamothioyl-furan-2-carboxamide derivatives

Muhammad Salman javed[1], Muhammad Zubair[1*], Komal Rizwan[2*], Muhammad Jamil[3]

[1]Department of Chemistry, Government College University Faisalabad, Faisalabad 38000, Pakistan

[2]Department of Chemistry, University of Sahiwal, Sahiwal, 57000, Pakistan

[3]Department of Chemistry, Government Post Graduate College, Sahiwal, Sahiwal 57000, Pakistan

*Corresponding authors: zubairmkn@gcuf.edu.pk (M. Zubair); komal.rizwan45@yahoo.com (K. Rizwan)

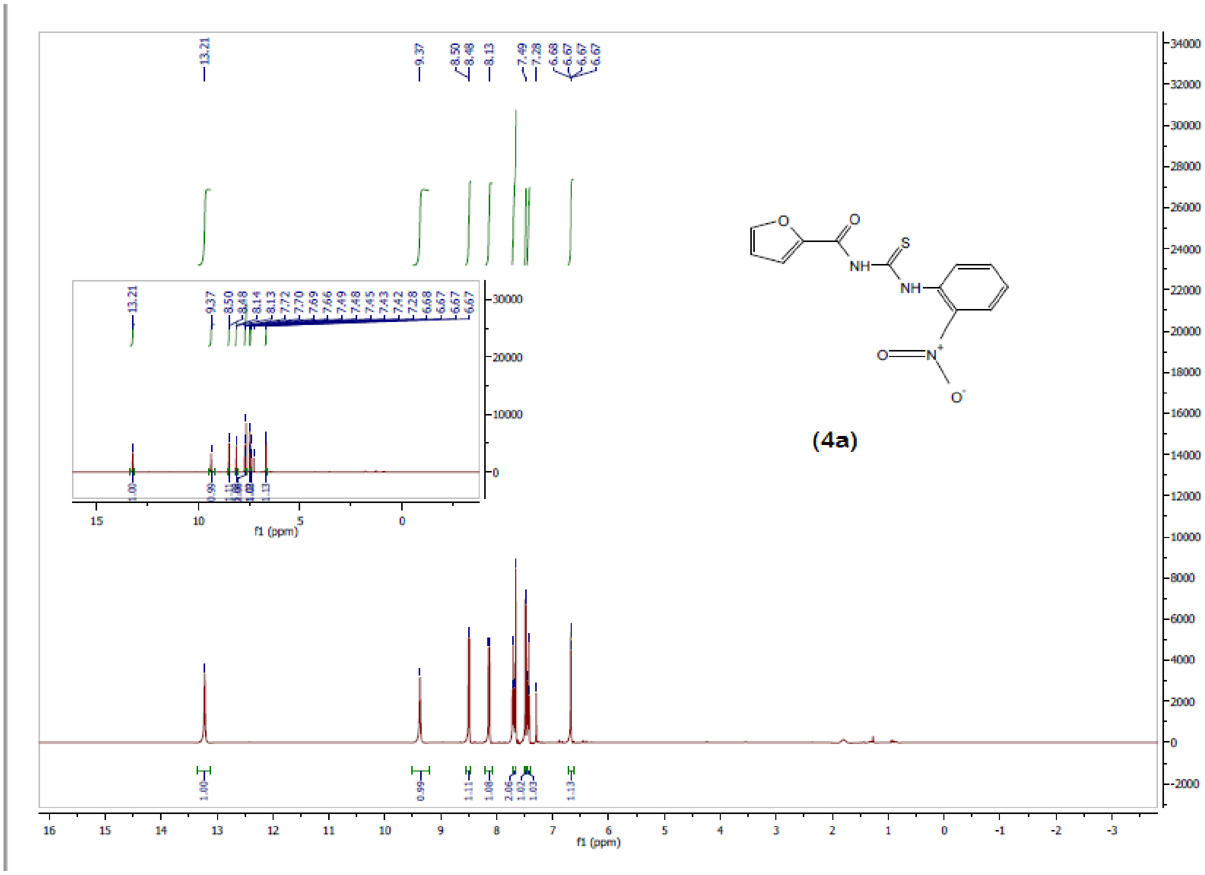

Proton NMR of 4a

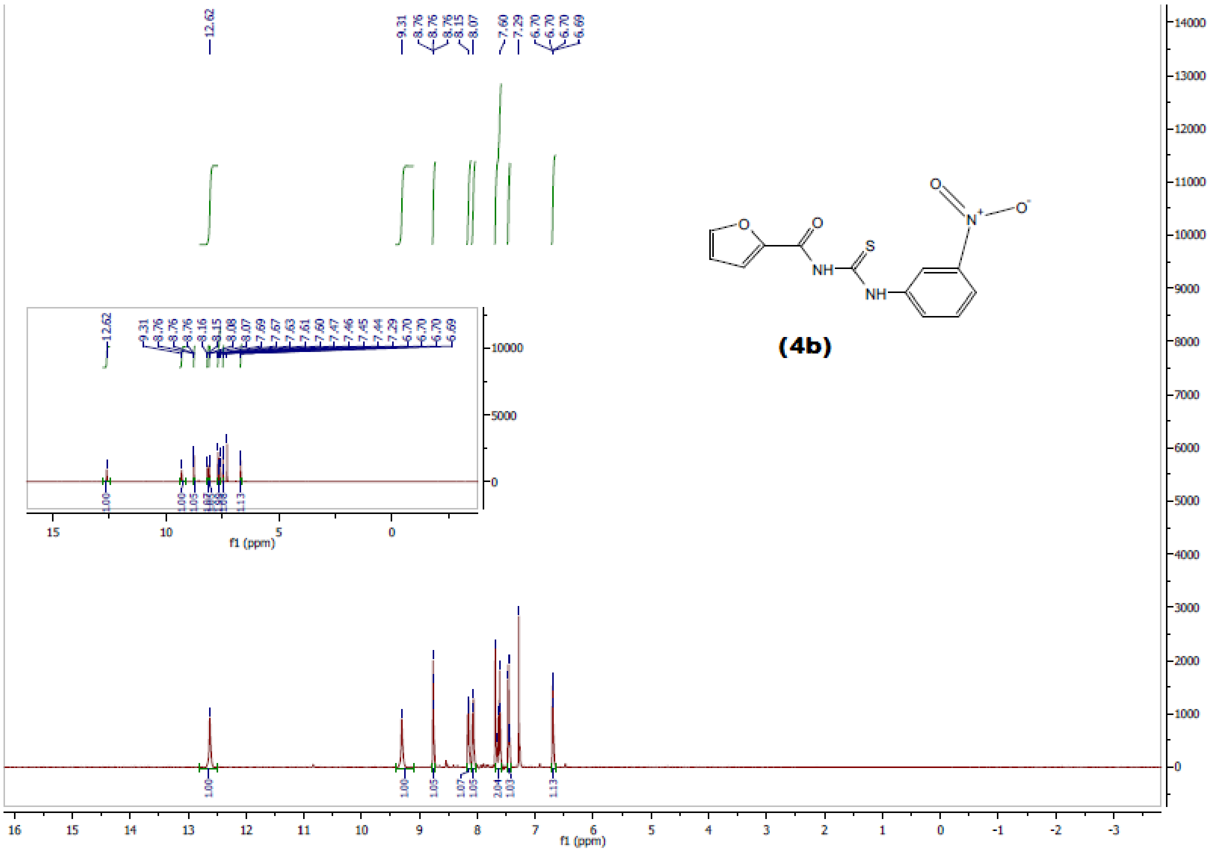

Proton NMR of 4b

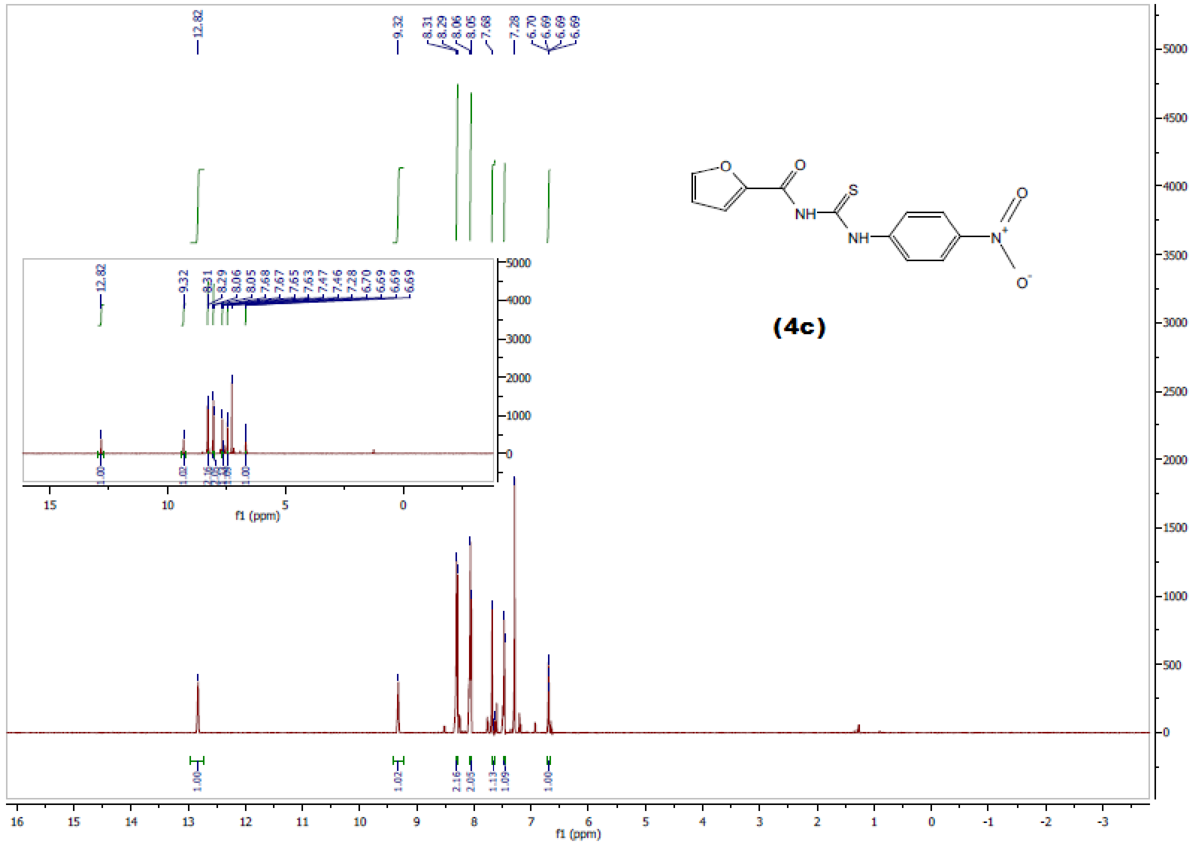

Proton NMR of 4c

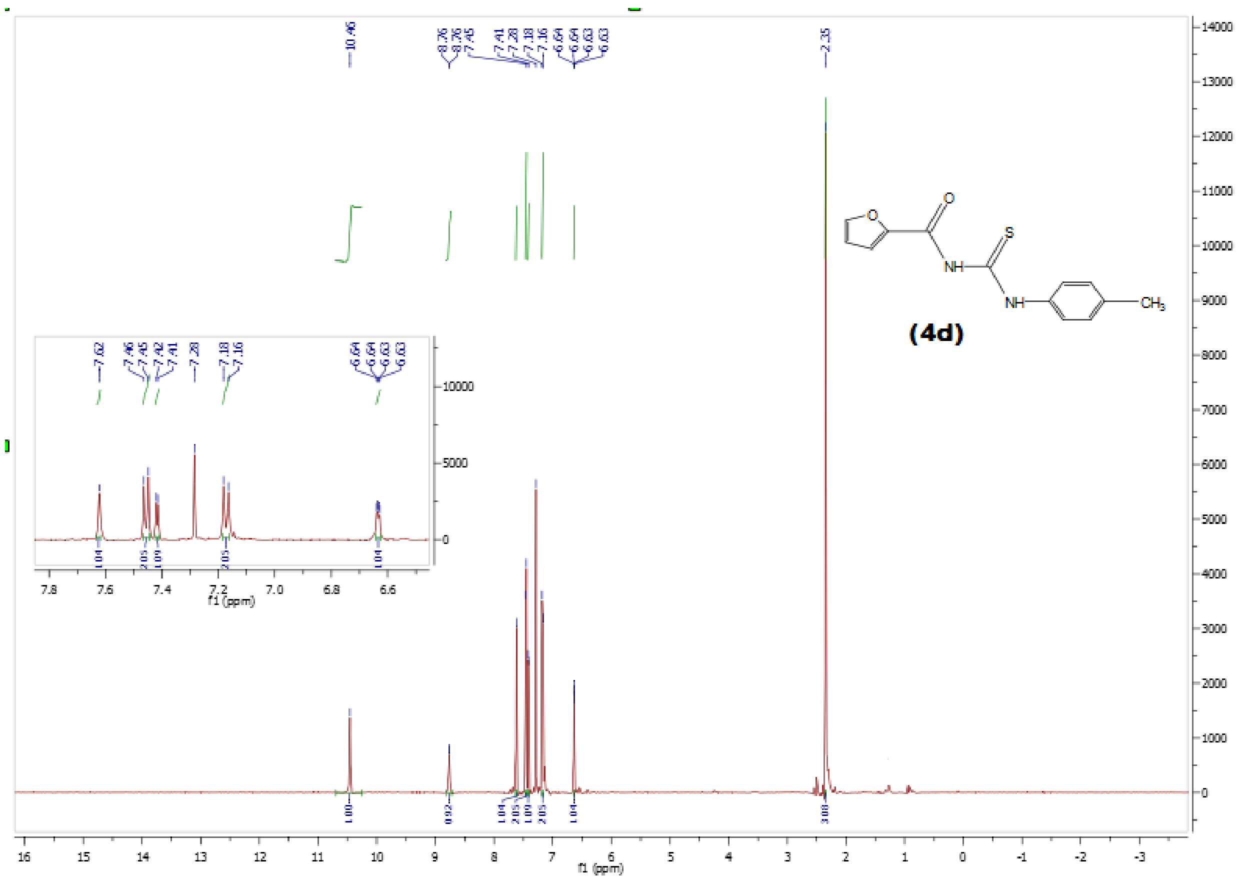

Proton NMR of 4d

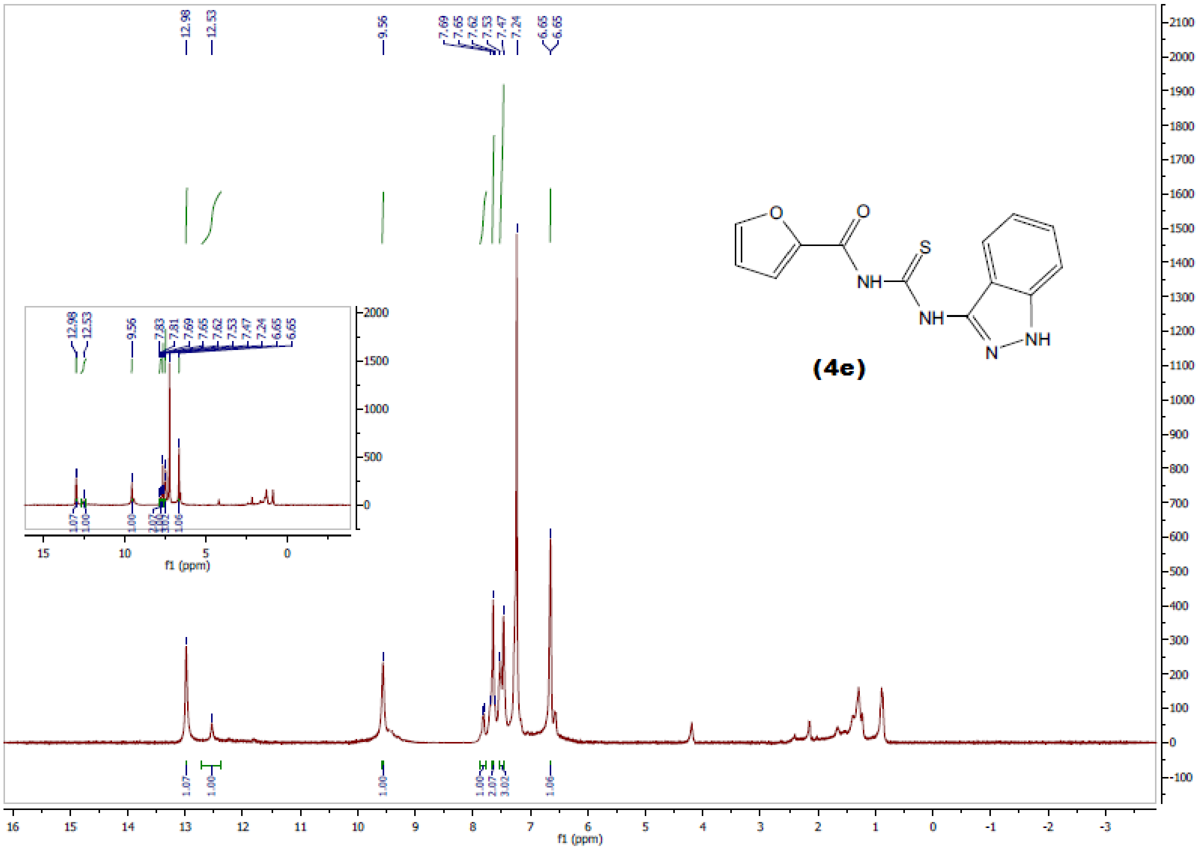

Proton NMR of 4e

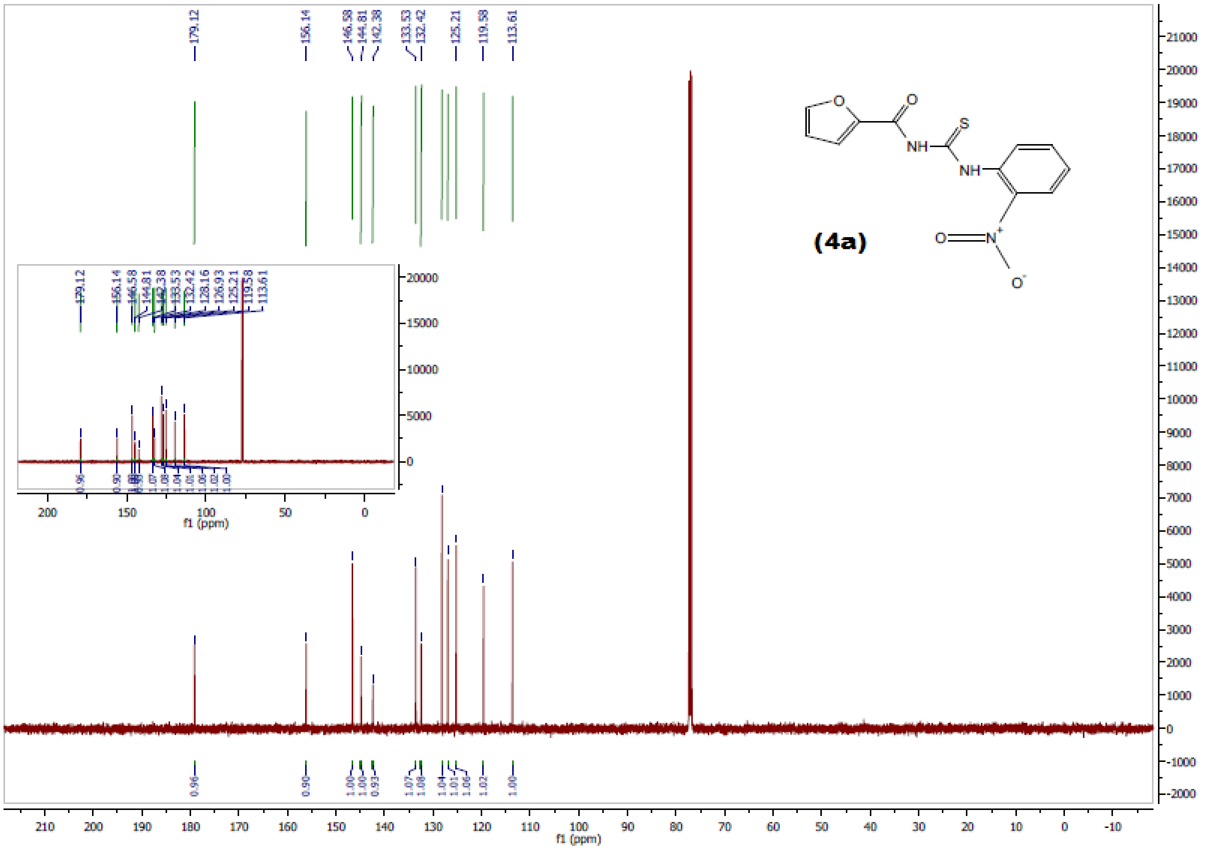

Carbon NMR of 4a

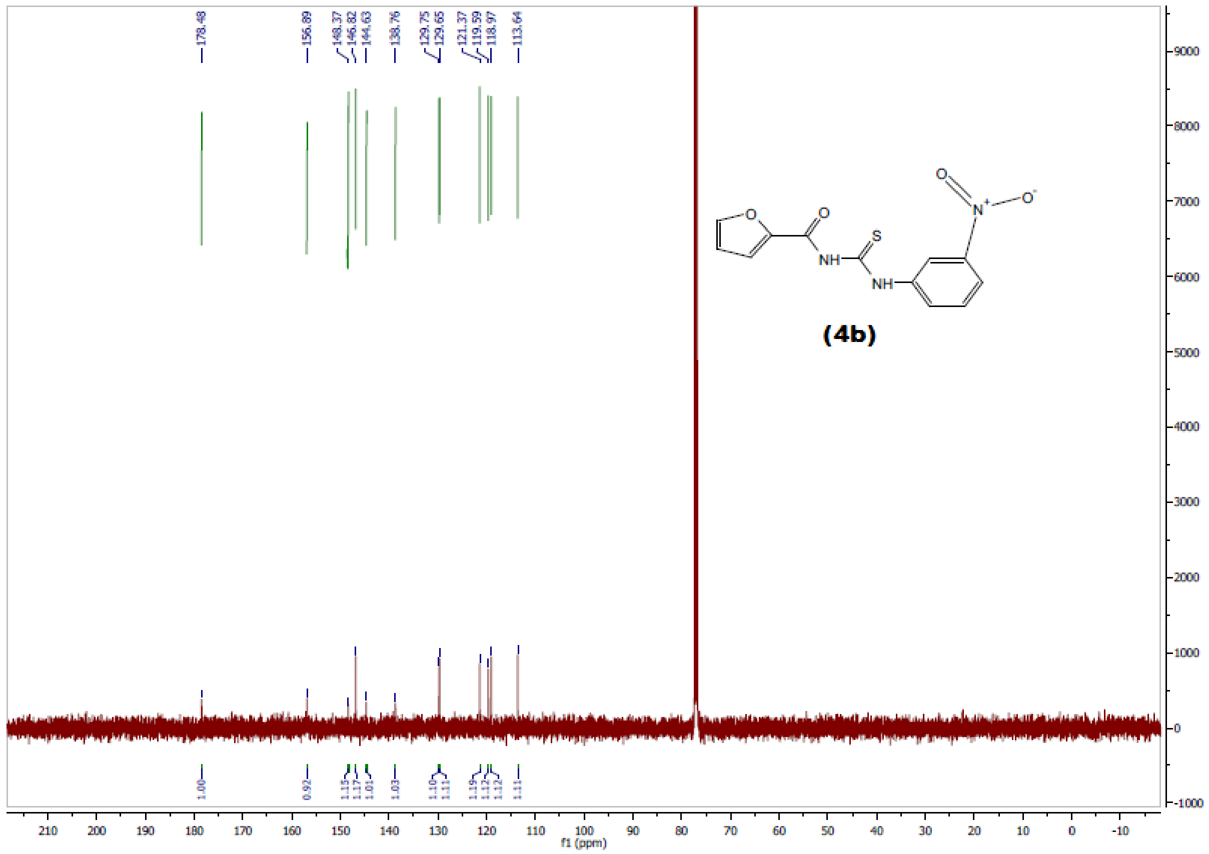

Carbon NMR of 4b

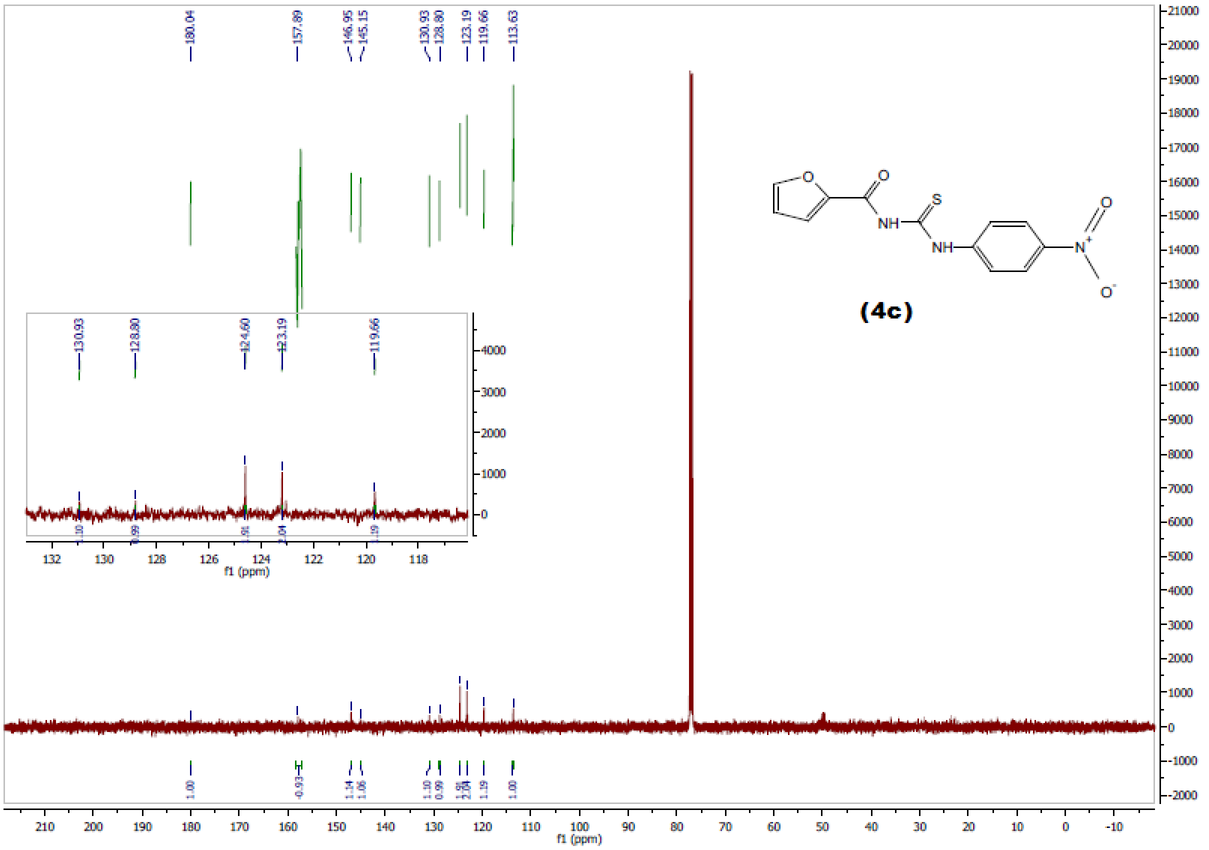

Carbon NMR of 4c

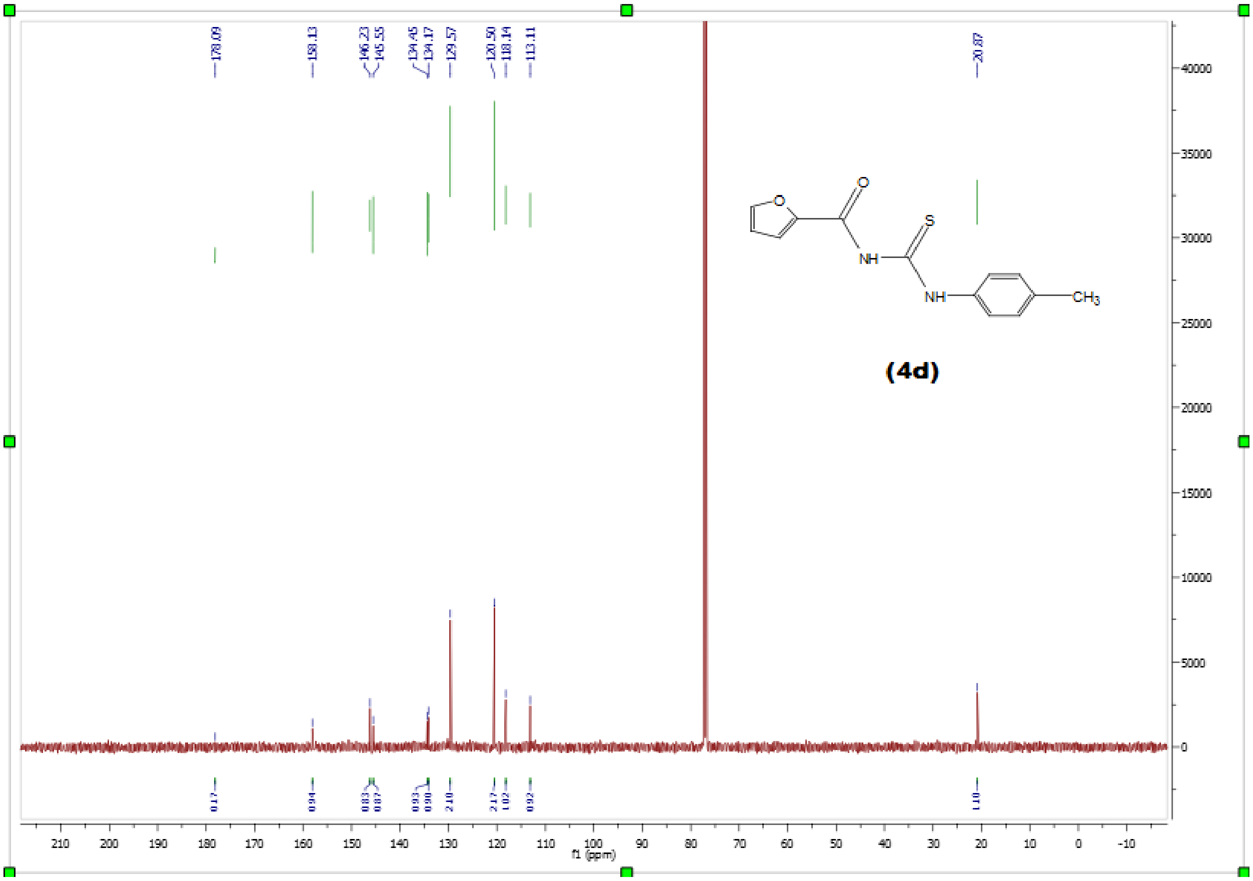

Carbon NMR of 4d

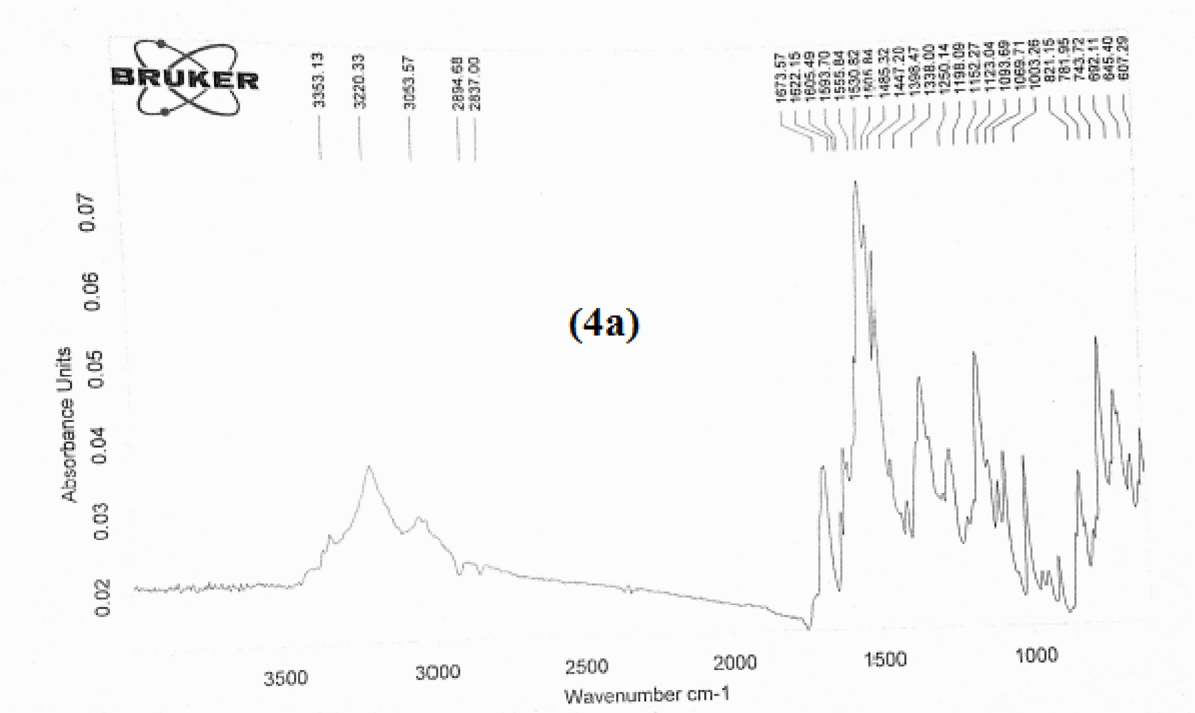

I.R spectrum of 4a

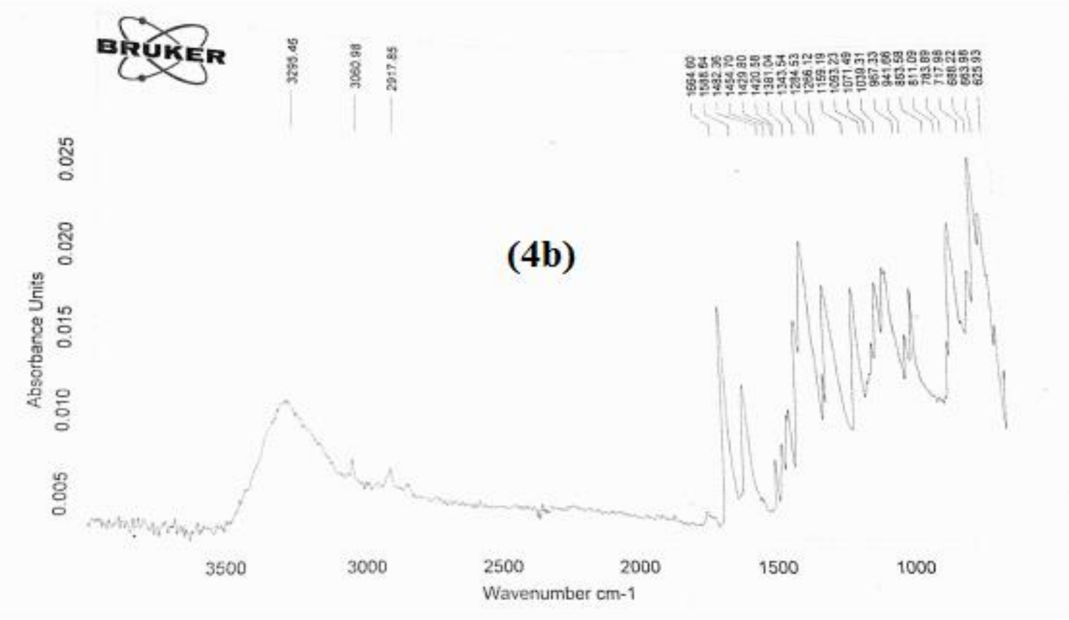

I.R spectrum of 4b

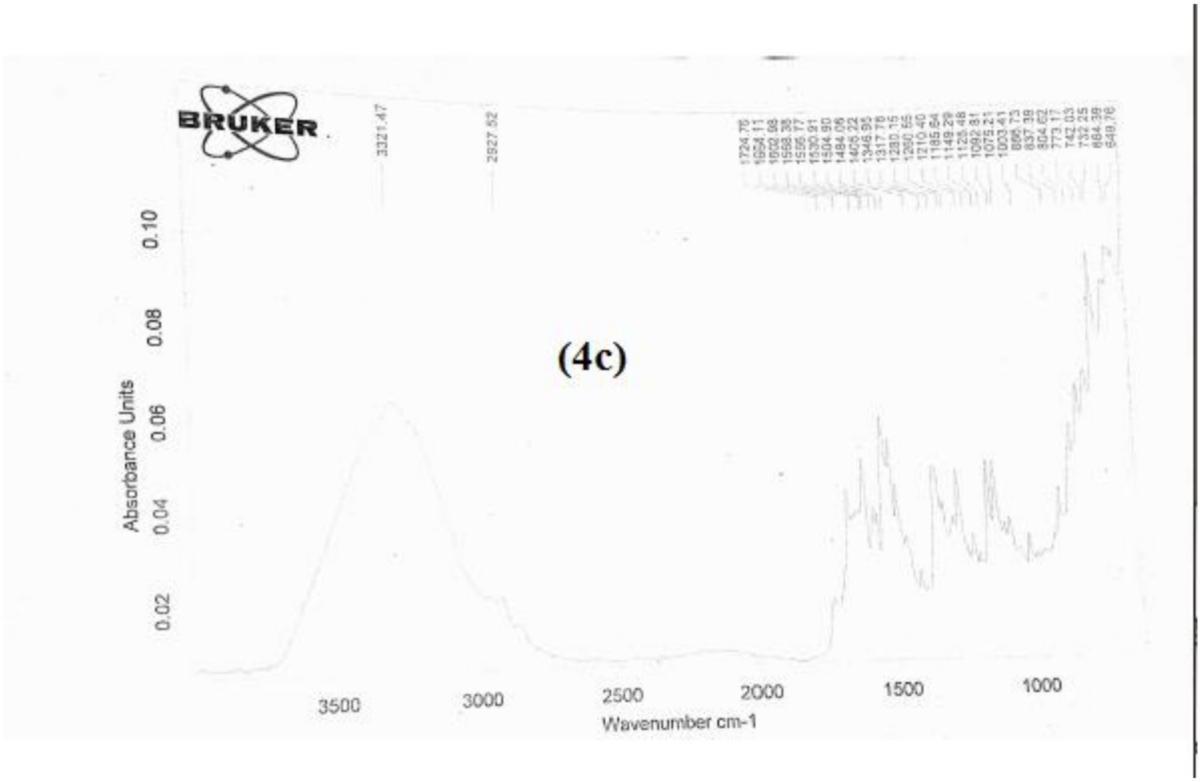

I.R spectrum of 4c

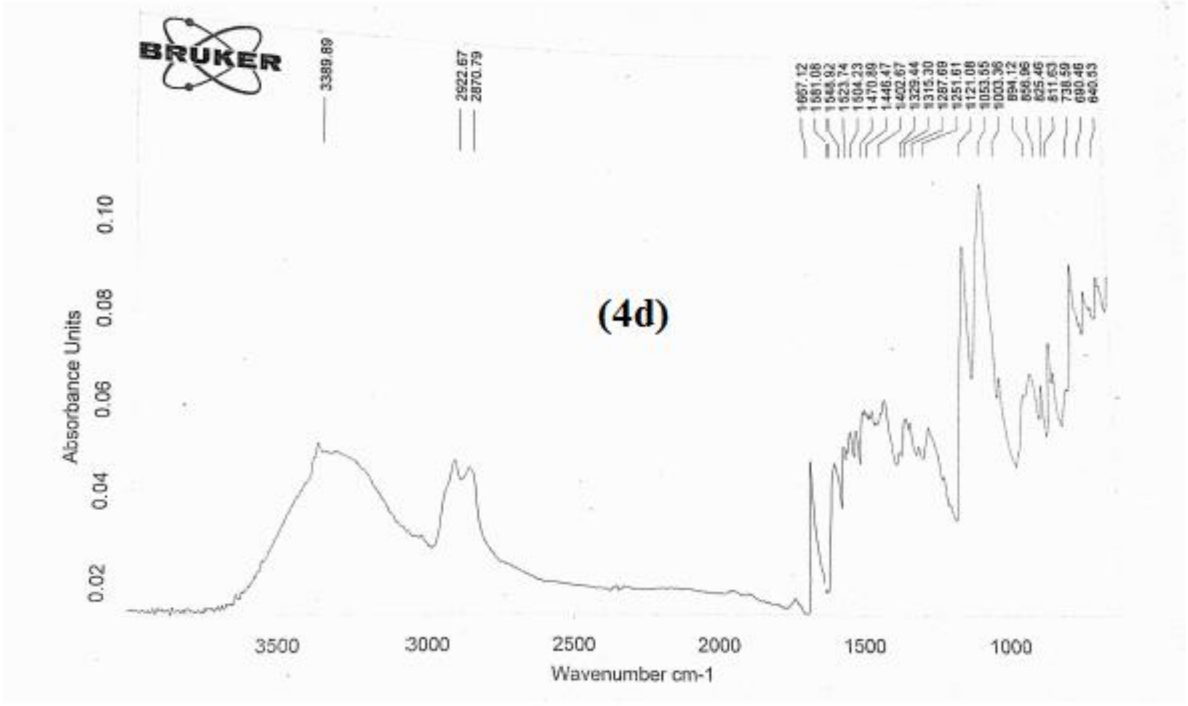

I.R spectrum of 4d

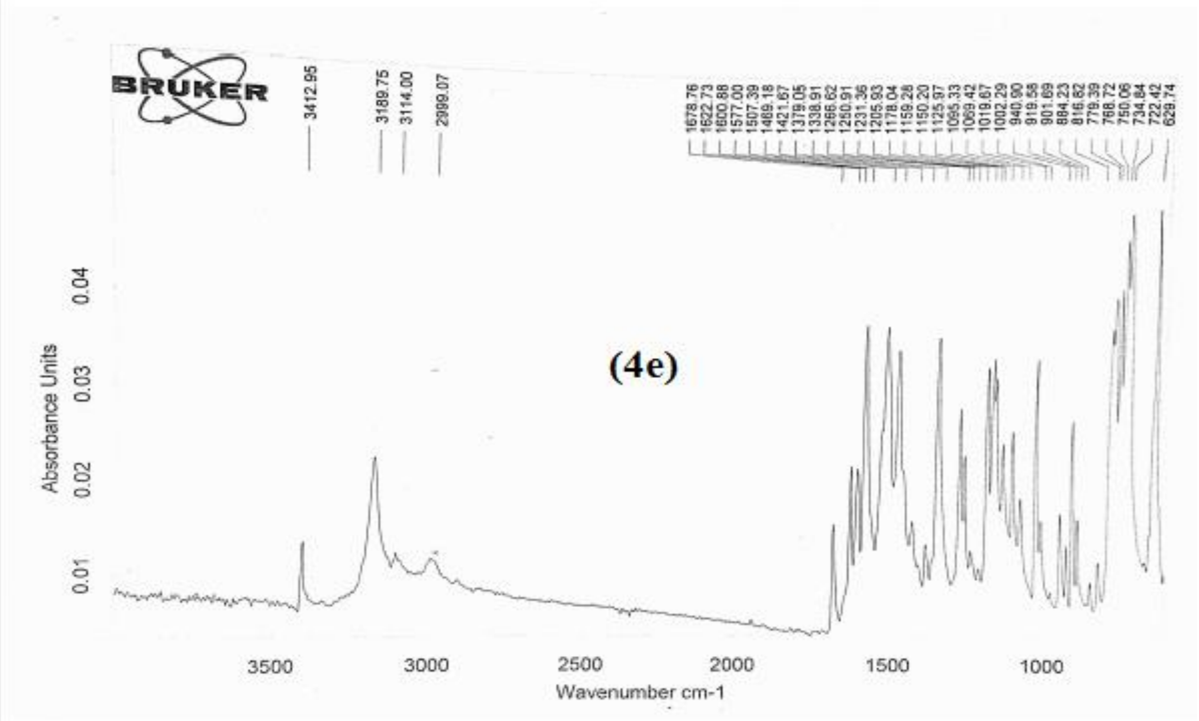

I.R spectrum of 4e

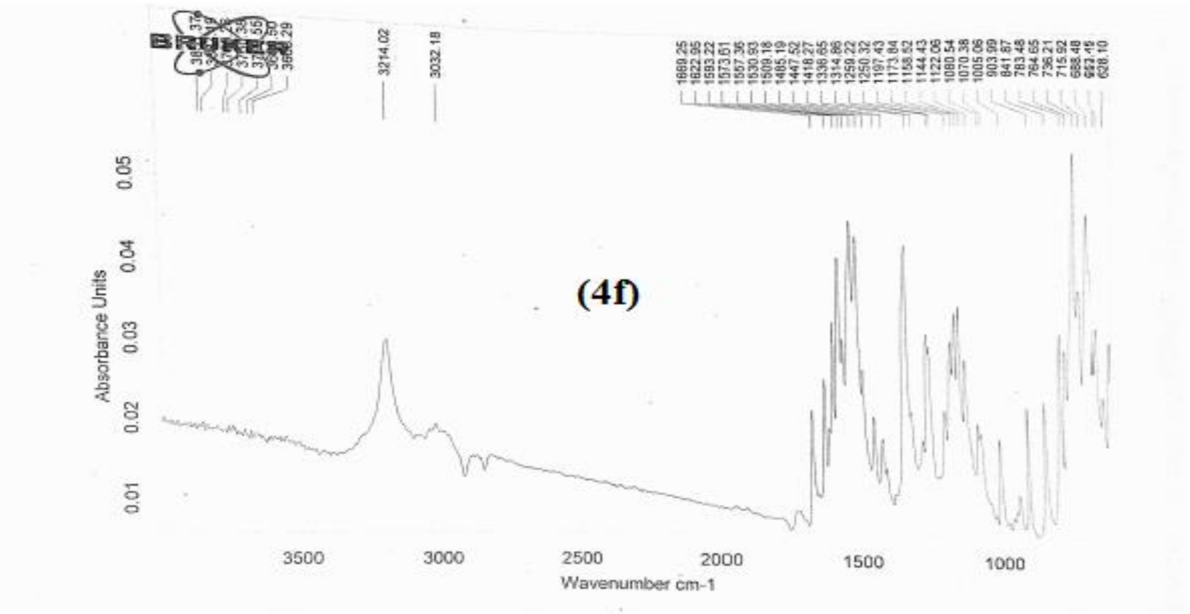

I.R spectrum of 4f

LC/MS Spectra of synthesized molecules

FULLSCAN

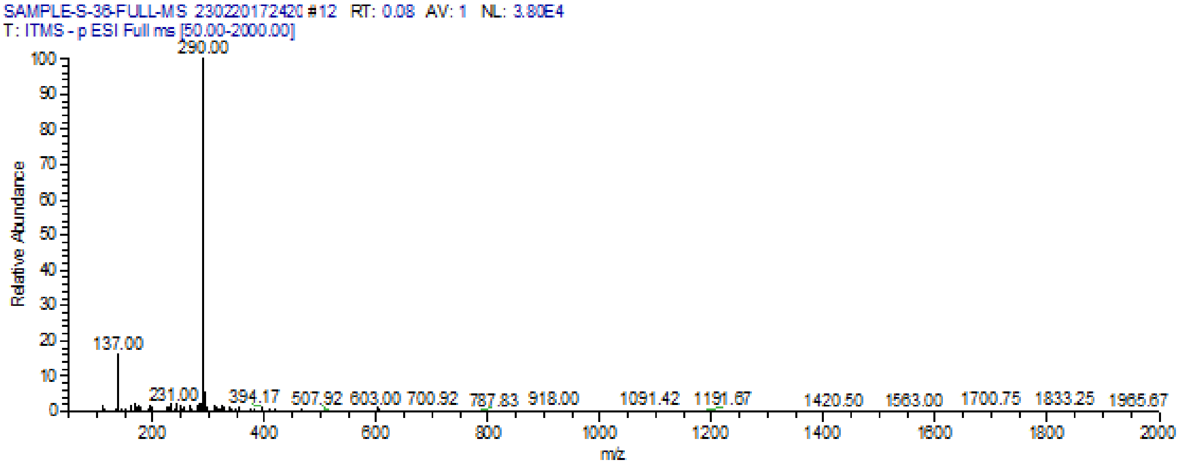

LC/MS Spectra of 4a

FULL SCAN

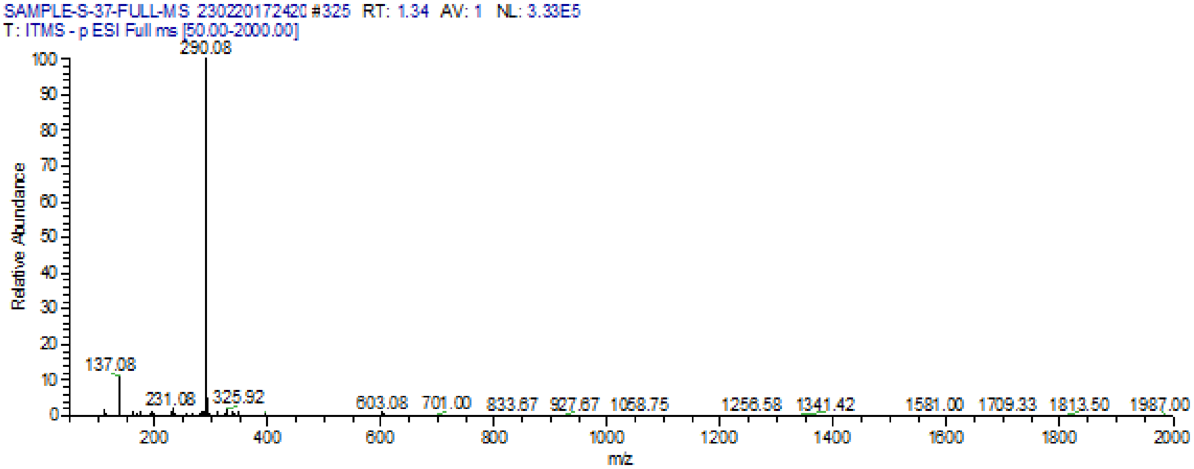

LC/MS Spectra of 4b

FULL SCAN

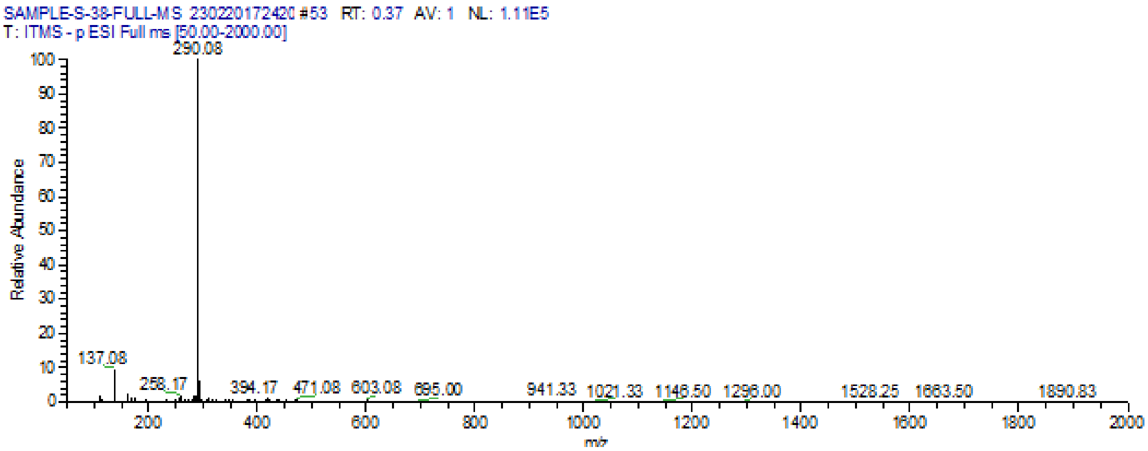

LC/MS Spectra of 4c

FULLSCAN

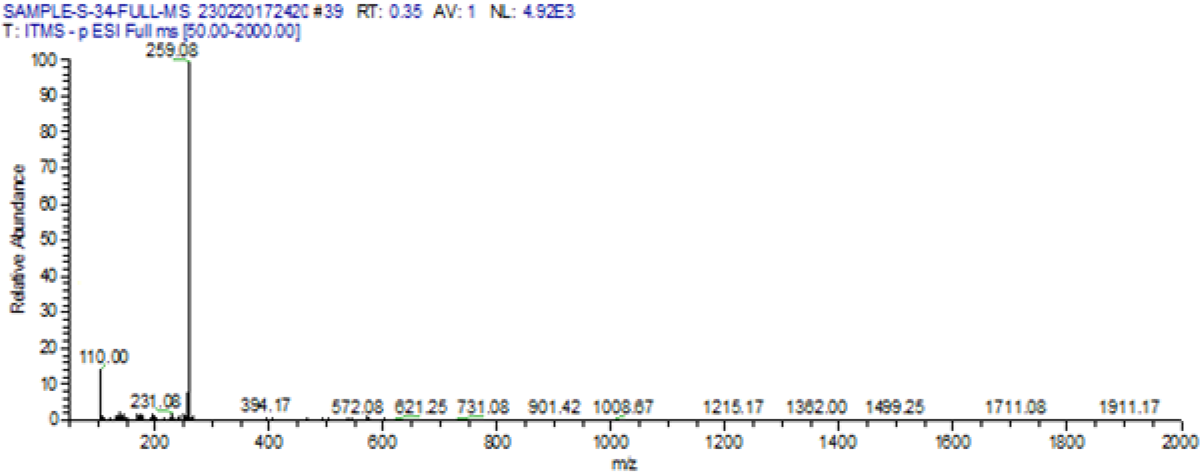

LC/MS Spectra of 4d

FULLSCAN

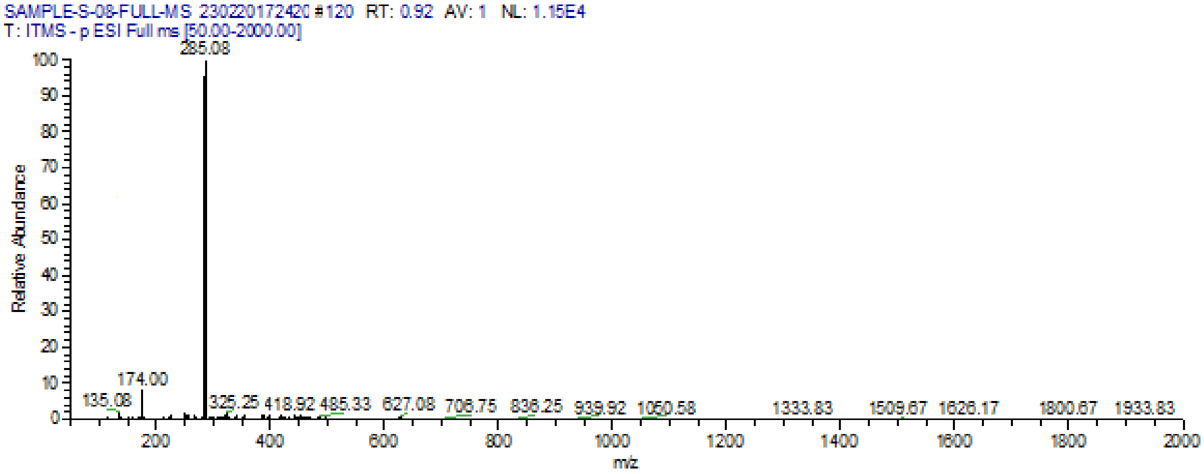

LC/MS Spectra of 4e

FULL SCAN

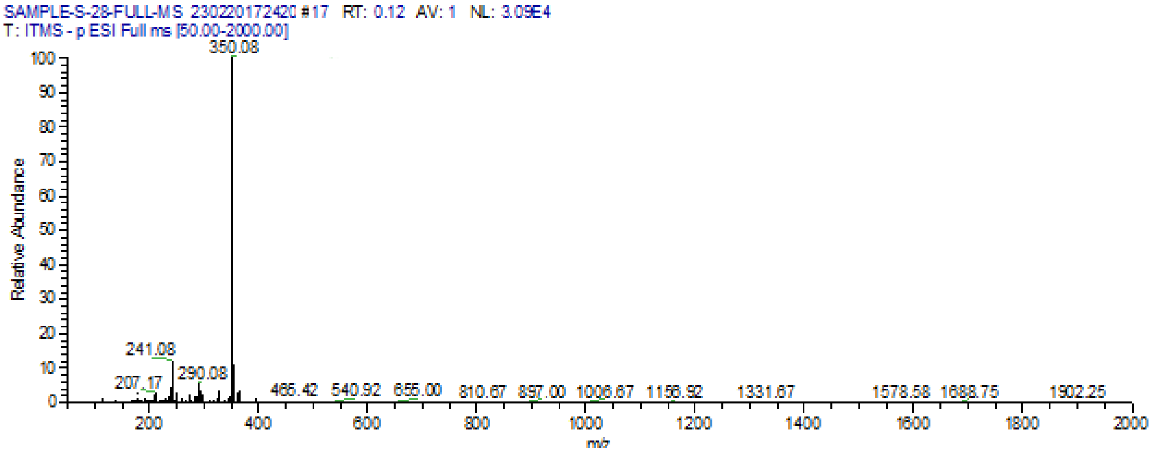

LC/MS Spectra of 4f

EI-HR-MS data of synthesized molecules

Instrument: JEOL JMS 600H-1
Inlet: Direct Probe
Ionization mode: EI+
Run By: MASS LAB 104

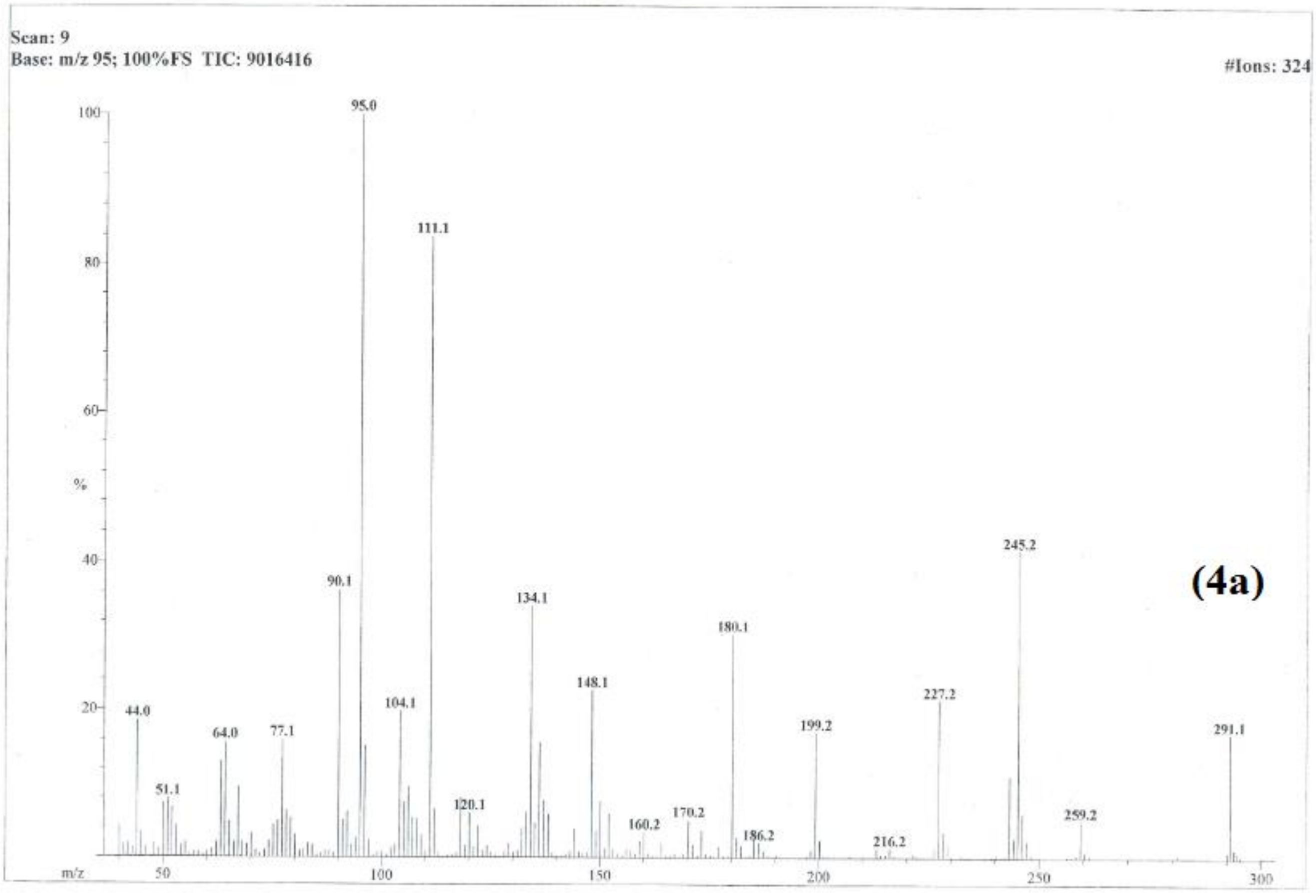

(4a)

Mass	Relative Intensity	Theoretical Mass	Delta [ppm]	Delta [mmu]	RDB	Composition
290.0161	0.1	290.0157	1.4	0.4	24.0	$C_{24} H_{2}$
		290.0150	3.7	1.1	15.0	$C_{16} H_{6} O_{2} N_{2} S_{1}$
		290.0190	-10.2	-3.0	19.0	$C_{21} H_{6} S_{1}$
		290.0202	-14.2	-4.1	15.5	$C_{15} H_{4} O_{4} N_{3}$
291.0297	1.9	291.0314	-5.8	-1.7	10.0	$C_{12} H_{9} O_{4} N_{3} S_{1}$
		291.0280	5.8	1.7	15.0	$C_{15} H_{5} O_{4} N_{3}$
		291.0320	-8.0	-2.3	19.0	$C_{20} H_{5} O_{2} N_{1}$
		291.0268	9.8	2.9	18.5	$C_{21} H_{7} S_{1}$

Instrument: JEOL JMS 600H-1
Inlet: Direct Probe

Ionization mode: EI+

Run By: MASS LAB 104

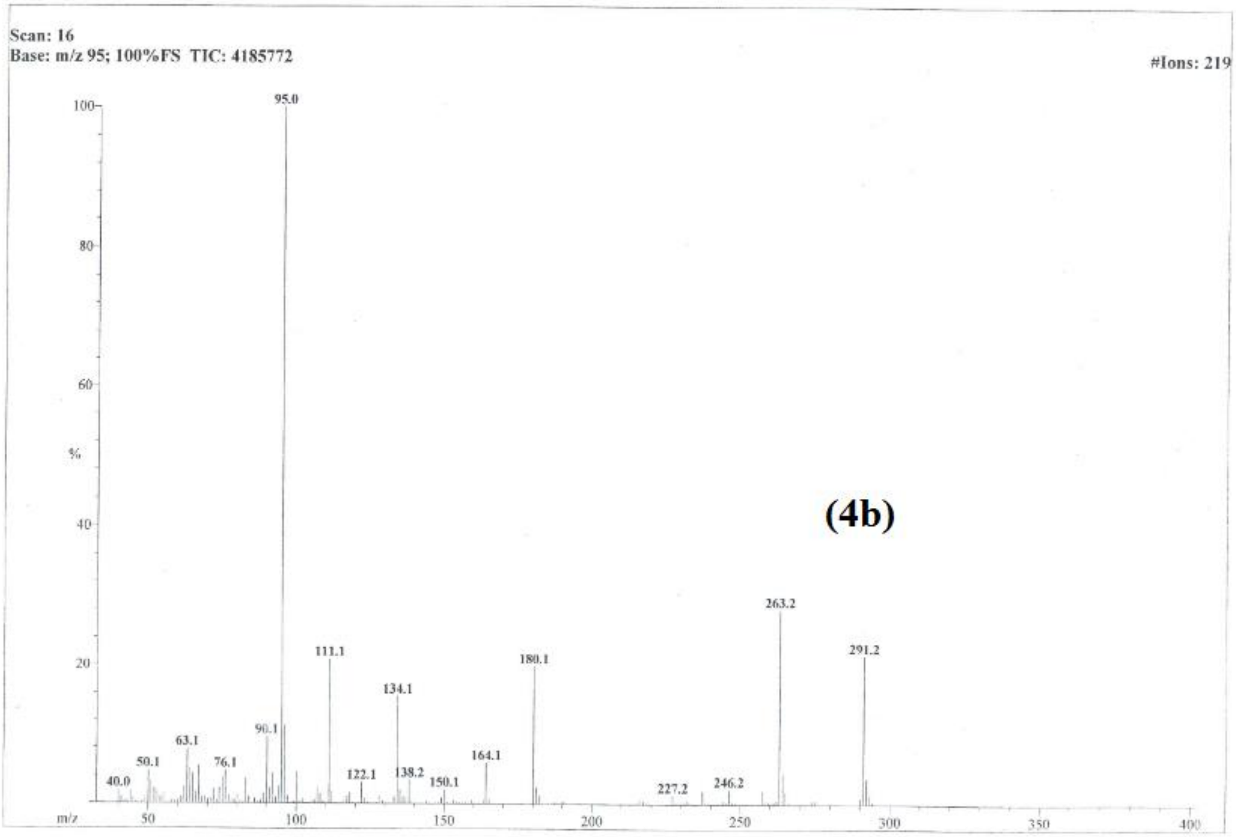

Mass	Relative Intensity	Theoretical Mass	Delta [ppm]	Delta [mmu]	RDB	Composition
		232.0181	-5.8	-1.3	9.5	$C_{10} H_6 O_2 N_3 S_1$
		232.0187	-8.6	-2.0	18.5	$C_{18} H_2 N_1$
		232.0147	8.8	2.0	14.5	$C_{13} H_2 O_2 N_3$
237.0129	0.8	237.0123	2.8	0.7	12.5	$C_{13} H_5 O_1 N_2 S_1$
		237.0096	14.1	3.3	8.0	$C_{10} H_7 O_4 N_1 S_1$
		237.0089	17.0	4.0	17.5	$C_{16} H_1 O_1 N_2$
		237.0174	-19.0	-4.5	13.0	$C_{12} H_3 O_3 N_3$
246.0340	0.6	246.0337	1.0	0.3	9.5	$C_{11} H_8 O_2 N_3 S_1$
		246.0344	-1.6	-0.4	18.5	$C_{19} H_4 N_1$
		246.0351	-4.4	-1.1	9.0	$C_{13} H_{10} O_3 S_1$
		246.0317	9.3	2.3	14.0	$C_{16} H_6 O_3$
263.0365	5.4	263.0365	0.3	0.1	9.0	$C_{11} H_9 O_3 N_3 S_1$
		263.0371	-2.1	-0.6	18.0	$C_{19} H_5 O_1 N_1$
		263.0378	-4.8	-1.3	8.5	$C_{13} H_{11} O_4 S_1$
		263.0344	8.0	2.1	13.5	$C_{16} H_7 O_4$
264.0375	0.7	264.0357	6.8	1.8	13.0	$C_{15} H_8 O_1 N_2 S_1$
		264.0409	-12.8	-3.4	13.5	$C_{14} H_6 O_3 N_3$
		264.0331	17.0	4.5	8.5	$C_{12} H_{10} O_4 N_1 S_1$
		264.0423	-17.9	-4.7	13.0	$C_{16} H_8 O_4$
279.1622	2.6	279.1623	-0.2	-0.1	10.0	$C_{19} H_{21} O_1 N_1$
		279.1617	2.1	0.6	1.0	$C_{11} H_{25} O_3 N_3 S_1$
		279.1630	-2.7	-0.8	0.5	$C_{13} H_{27} O_4 S_1$
		279.1596	9.4	2.6	5.5	$C_{16} H_{23} O_4$
291.0326	3.7	291.0320	2.0	0.6	19.0	$C_{20} H_5 O_2 N_1$
		291.0314	4.3	1.2	10.0	$C_{12} H_9 O_4 N_3 S_1$
		291.0354	-9.5	-2.8	14.0	$C_{17} H_9 O_2 N_1 S_1$
		291.0280	15.9	4.6	15.0	$C_{15} H_5 O_4 N_3$
292.0261	0.5	292.0273	-4.0	-1.2	19.0	$C_{19} H_4 O_2 N_2$
		292.0221	13.8	4.0	18.5	$C_{20} H_6 N_1 S_1$
		292.0306	-15.5	-4.5	14.0	$C_{16} H_8 O_2 N_2 S_1$
		292.0313	-17.7	-5.2	23.0	$C_{24} H_4$

Instrument: JEOL JMS 600H-1
Inlet: Direct Probe

Ionization mode: EI+

Run By: MASS LAB 104

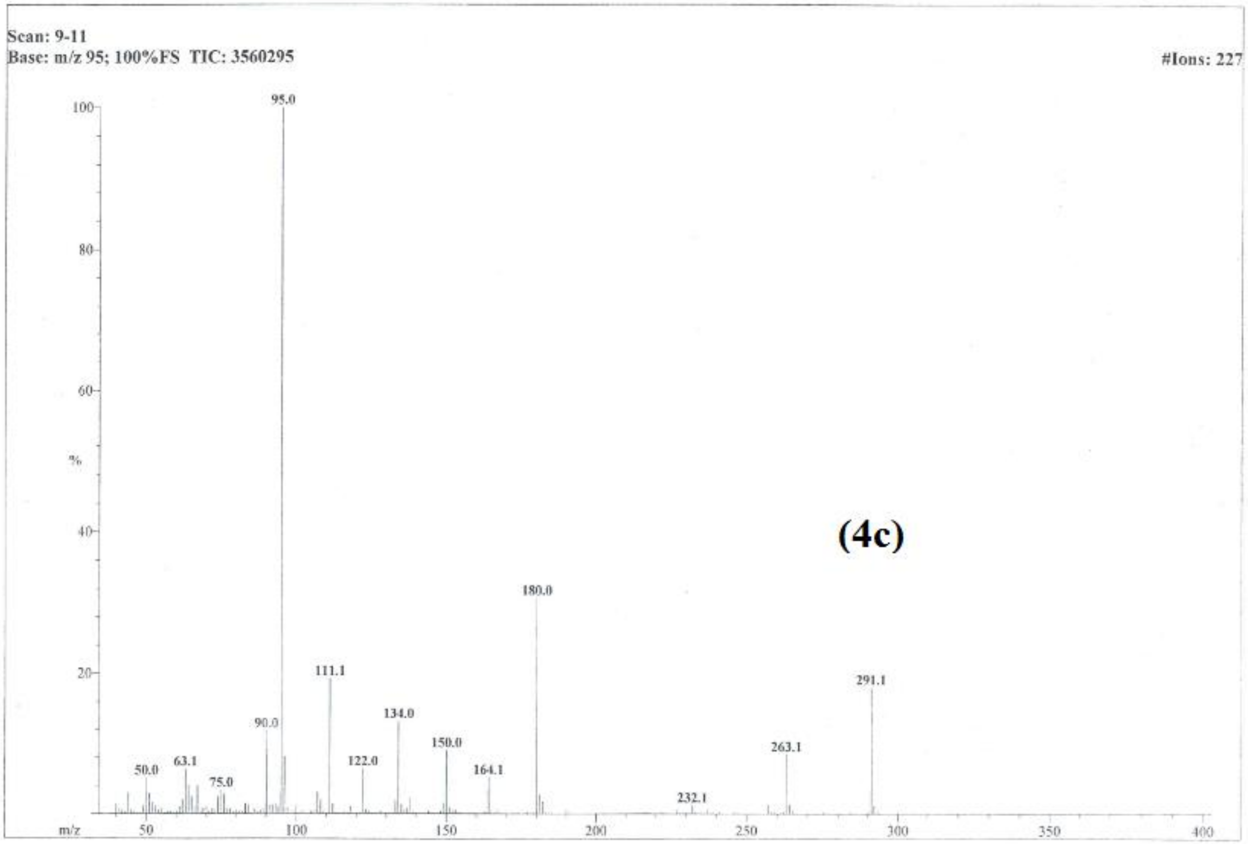

Mass	Relative Intensity	Theoretical Mass	Delta [ppm]	Delta [mmu]	RDB	Composition
		232.0518	-18.5	-4.3	4.0	$C_8 H_{12} O_4 N_2 S_1$
		232.0524	-21.3	-4.9	13.0	$C_{16} H_8 O_2$
		232.0545	-30.0	-7.0	8.5	$C_{11} H_{10} O_1 N_3 S_1$
263.0362	8.9	263.0365	-1.2	-0.3	9.0	$C_{11} H_9 O_3 N_3 S_1$
		263.0371	-3.6	-1.0	18.0	$C_{19} H_5 O_1 N_1$
		263.0378	-6.3	-1.6	8.5	$C_{13} H_{11} O_4 S_1$
		263.0344	6.5	1.7	13.5	$C_{16} H_7 O_4$
		263.0331	11.6	3.1	14.0	$C_{14} H_5 O_3 N_3$
		263.0405	-16.5	-4.3	13.0	$C_{16} H_9 O_1 N_1 S_1$
264.0365	1.3	264.0357	2.7	0.7	13.0	$C_{15} H_8 O_1 N_2 S_1$
		264.0331	12.9	3.4	8.5	$C_{12} H_{10} O_4 N_1 S_1$
		264.0324	15.5	4.1	18.0	$C_{18} H_4 O_1 N_2$
		264.0409	-16.9	-4.5	13.5	$C_{14} H_6 O_3 N_3$
		264.0423	-22.0	-5.8	13.0	$C_{16} H_8 O_4$
		264.0297	25.7	6.8	13.5	$C_{15} H_6 O_4 N_1$
291.0297	6.8	291.0314	-5.6	-1.6	10.0	$C_{12} H_9 O_4 N_3 S_1$
		291.0280	5.9	1.7	15.0	$C_{15} H_5 O_4 N_3$
		291.0320	-7.9	-2.3	19.0	$C_{20} H_5 O_2 N_1$
		291.0268	9.9	2.9	18.5	$C_{21} H_7 S_1$
		291.0354	-19.5	-5.7	14.0	$C_{17} H_9 O_2 N_1 S_1$
		291.0235	21.5	6.3	23.5	$C_{24} H_3$
292.0332	1.0	292.0347	-5.0	-1.5	18.0	$C_{21} H_8 S_1$
		292.0313	6.5	1.9	23.0	$C_{24} H_4$
		292.0306	8.8	2.6	14.0	$C_{16} H_8 O_2 N_2 S_1$
		292.0358	-9.0	-2.6	14.5	$C_{15} H_6 O_4 N_3$
		292.0273	20.3	5.9	19.0	$C_{19} H_4 O_2 N_2$
		292.0392	-20.5	-6.0	9.5	$C_{12} H_{10} O_4 N_3 S_1$

Instrument: JEOL JMS 600H-1
Inlet: Direct Probe

Ionization mode: EI+

Run By: MASS LAB 104

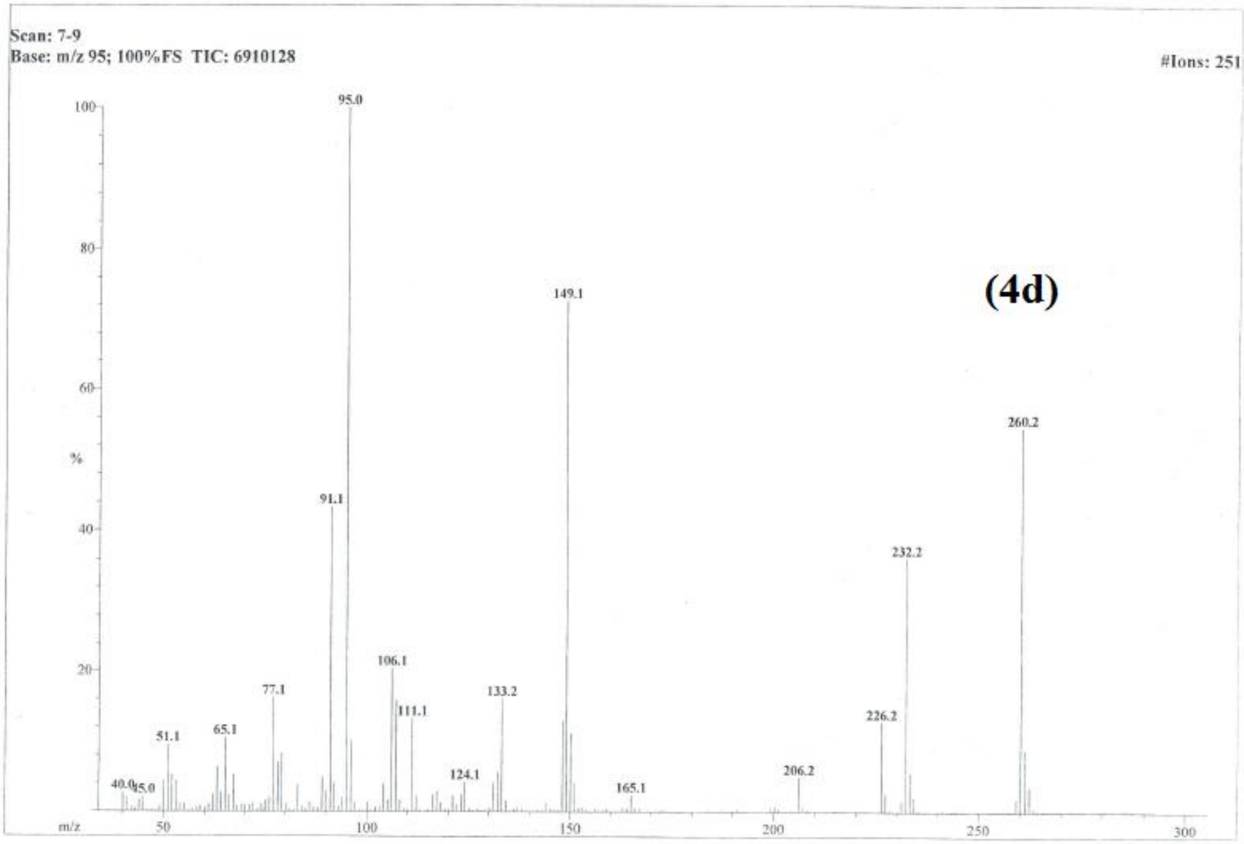

Mass	Relative Intensity	Theoretical Mass	Delta [ppm]	Delta [mmu]	RDB	Composition
		150.0344	-16.7	-2.5	10.5	$C_{11} H_{4} N_{1}$
		150.0351	-21.3	-3.2	1.0	$C_{5} H_{10} O_{3} S_{1}$
		150.0377	-39.1	-5.9	5.5	$C_{8} H_{8} N_{1} S_{1}$
151.0237	1.4	151.0218	13.0	2.0	5.5	$C_{8} H_{7} O_{1} S_{1}$
		151.0269	-21.3	-3.2	6.0	$C_{7} H_{5} O_{3} N_{1}$
		151.0184	35.3	5.3	10.5	$C_{11} H_{3} O_{1}$
		151.0296	-39.0	-5.9	10.5	$C_{10} H_{3} N_{2}$
200.0937	1.4	200.0950	-6.5	-1.3	8.0	$C_{12} H_{12} O_{1} N_{2}$
		200.0923	6.9	1.4	3.5	$C_{9} H_{14} O_{4} N_{1}$
		200.0983	-23.3	-4.7	3.0	$C_{9} H_{16} O_{1} N_{2} S_{1}$
		200.0871	32.8	6.6	3.0	$C_{10} H_{16} O_{2} S_{1}$
206.0502	1.1	206.0514	-5.6	-1.1	7.0	$C_{10} H_{10} O_{1} N_{2} S_{1}$
		206.0487	7.4	1.5	2.5	$C_{7} H_{12} O_{4} N_{1} S_{1}$
		206.0480	10.8	2.2	12.0	$C_{13} H_{6} O_{1} N_{2}$
		206.0453	23.8	4.9	7.5	$C_{10} H_{8} O_{4} N_{1}$
226.0741	6.4	226.0742	-0.7	-0.2	10.0	$C_{13} H_{10} O_{2} N_{2}$
		226.0776	-15.6	-3.5	5.0	$C_{10} H_{14} O_{2} N_{2} S_{1}$
		226.0783	-18.5	-4.2	14.0	$C_{18} H_{10}$
		226.0690	22.2	5.0	9.5	$C_{14} H_{12} N_{1} S_{1}$
227.0740	1.5	227.0742	-0.8	-0.2	4.5	$C_{11} H_{15} O_{3} S_{1}$
		227.0735	2.3	0.5	14.0	$C_{17} H_{9} N_{1}$
		227.0769	-12.6	-2.9	9.0	$C_{14} H_{13} N_{1} S_{1}$
		227.0708	14.1	3.2	9.5	$C_{14} H_{11} O_{3}$
232.0649	9.7	232.0644	2.5	0.6	3.5	$C_{9} H_{14} O_{4} N_{1} S_{1}$
		232.0637	5.4	1.3	13.0	$C_{15} H_{8} O_{1} N_{2}$
		232.0670	-9.1	-2.1	8.0	$C_{12} H_{12} O_{1} N_{2} S_{1}$
		232.0610	17.0	3.9	8.5	$C_{12} H_{10} O_{4} N_{1}$
233.0688	1.4	233.0688	0.0	0.0	8.0	$C_{12} H_{11} O_{4} N_{1}$
		233.0715	-11.5	-2.7	12.5	$C_{15} H_{9} O_{1} N_{2}$
		233.0722	-14.4	-3.4	3.0	$C_{9} H_{15} O_{4} N_{1} S_{1}$
		233.0636	22.3	5.2	7.5	$C_{13} H_{13} O_{2} S_{1}$
260.0624	13.1	260.0626	-0.6	-0.2	18.0	$C_{21} H_{8}$
		260.0619	1.9	0.5	9.0	$C_{13} H_{12} O_{2} N_{2} S_{1}$
		260.0660	-13.6	-3.5	13.0	$C_{18} H_{12} S_{1}$
		260.0586	14.8	3.9	14.0	$C_{16} H_{8} O_{2} N_{2}$
261.0678	2.1	261.0664	5.2	1.4	13.5	$C_{16} H_{9} O_{2} N_{2}$
		261.0698	-7.7	-2.0	8.5	$C_{13} H_{13} O_{2} N_{2} S_{1}$
		261.0704	-10.2	-2.7	17.5	$C_{21} H_{9}$
		261.0738	-23.1	-6.0	12.5	$C_{18} H_{13} S_{1}$

Instrument: JEOL JMS 600H-1
Inlet: Direct Probe

Ionization mode: EI+

Run By: MASS LAB 104

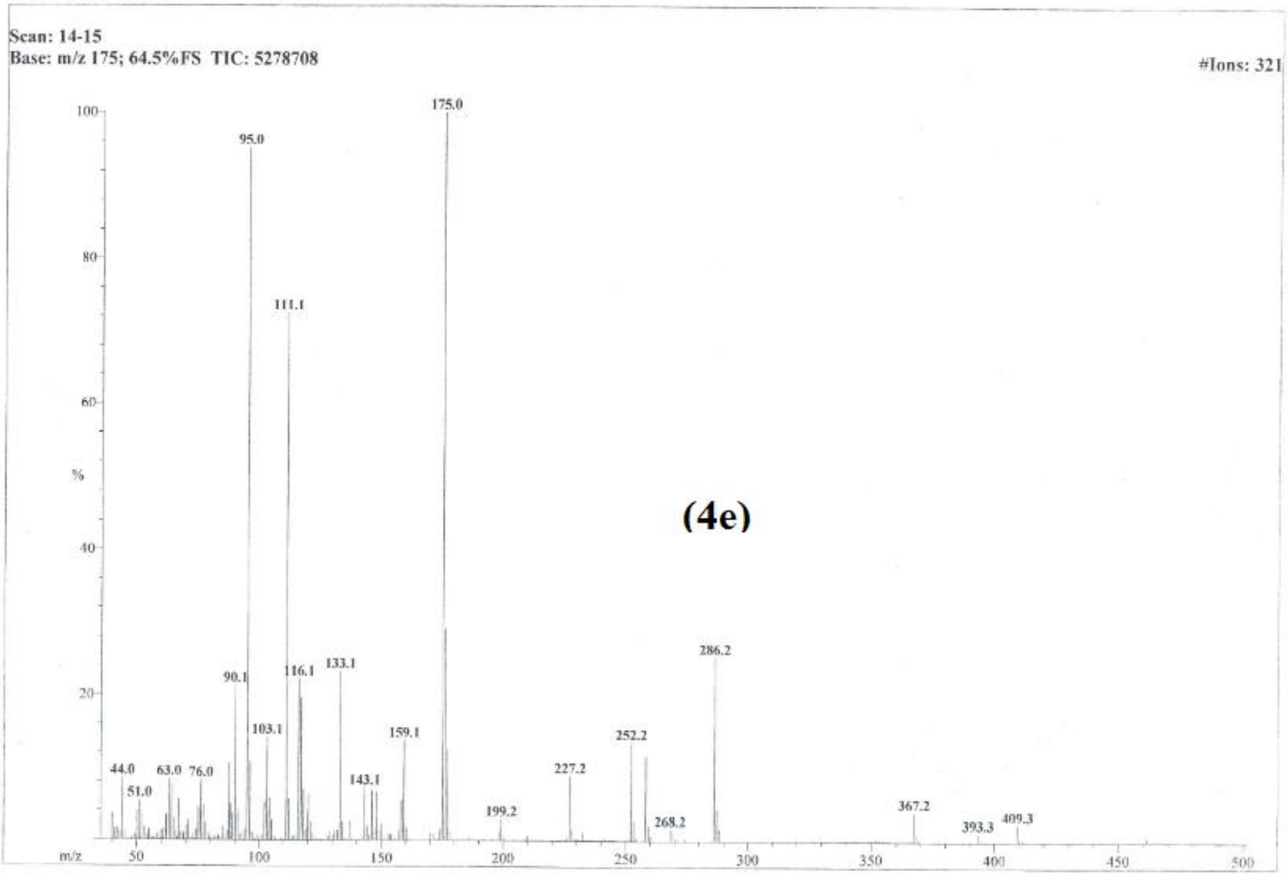

Mass	Relative Intensity	Theoretical Mass	Delta [ppm]	Delta [mmu]	RDB	Composition
		262.1477	10.1	2.6	1.5	$C_{12} H_{24} O_3 N_1 S_1$
		262.1470	12.7	3.3	11.0	$C_{18} H_{18} N_2$
		262.1463	15.2	4.0	2.0	$C_{10} H_{22} O_2 N_4 S_1$
279.1571	45.5	279.1583	-4.3	-1.2	6.0	$C_{14} H_{21} O_3 N_3$
		279.1610	-13.9	-3.9	10.5	$C_{17} H_{19} N_4$
		279.1531	14.3	4.0	5.5	$C_{15} H_{23} O_1 N_2 S_1$
		279.1617	-16.4	-4.6	1.0	$C_{11} H_{25} O_3 N_3 S_1$
280.1637	7.0	280.1661	-8.7	-2.4	5.5	$C_{14} H_{22} O_3 N_3$
		280.1609	9.8	2.7	5.0	$C_{15} H_{24} O_1 N_2 S_1$
		280.1688	-18.3	-5.1	10.0	$C_{17} H_{20} N_4$
		280.1695	-20.7	-5.8	0.5	$C_{11} H_{26} O_3 N_3 S_1$
281.1651	1.0	281.1654	-1.0	-0.3	9.5	$C_{18} H_{21} O_1 N_2$
		281.1647	1.3	0.4	0.5	$C_{10} H_{25} O_3 N_4 S_1$
		281.1688	-13.0	-3.6	4.5	$C_{15} H_{25} O_1 N_2 S_1$
		281.1614	13.3	3.7	5.5	$C_{13} H_{21} O_3 N_4$
286.0531	3.7	286.0531	-0.0	-0.0	20.0	$C_{21} H_6 N_2$
		286.0524	2.3	0.6	11.0	$C_{13} H_{10} O_2 N_4 S_1$
		286.0538	-2.4	-0.7	10.5	$C_{15} H_{12} O_3 N_1 S_1$
		286.0504	9.4	2.7	15.5	$C_{18} H_8 O_3 N_1$
287.0602	0.6	287.0603	-0.1	-0.0	10.5	$C_{13} H_{11} O_2 N_4 S_1$
		287.0609	-2.3	-0.7	19.5	$C_{21} H_7 N_2$
		287.0616	-4.8	-1.4	10.0	$C_{15} H_{13} O_3 N_1 S_1$
		287.0582	7.0	2.0	15.0	$C_{18} H_9 O_3 N_1$

Instrument: JEOL JMS 600H-1
Inlet: Direct Probe

Ionization mode: EI+

Run By: MASS LAB 104

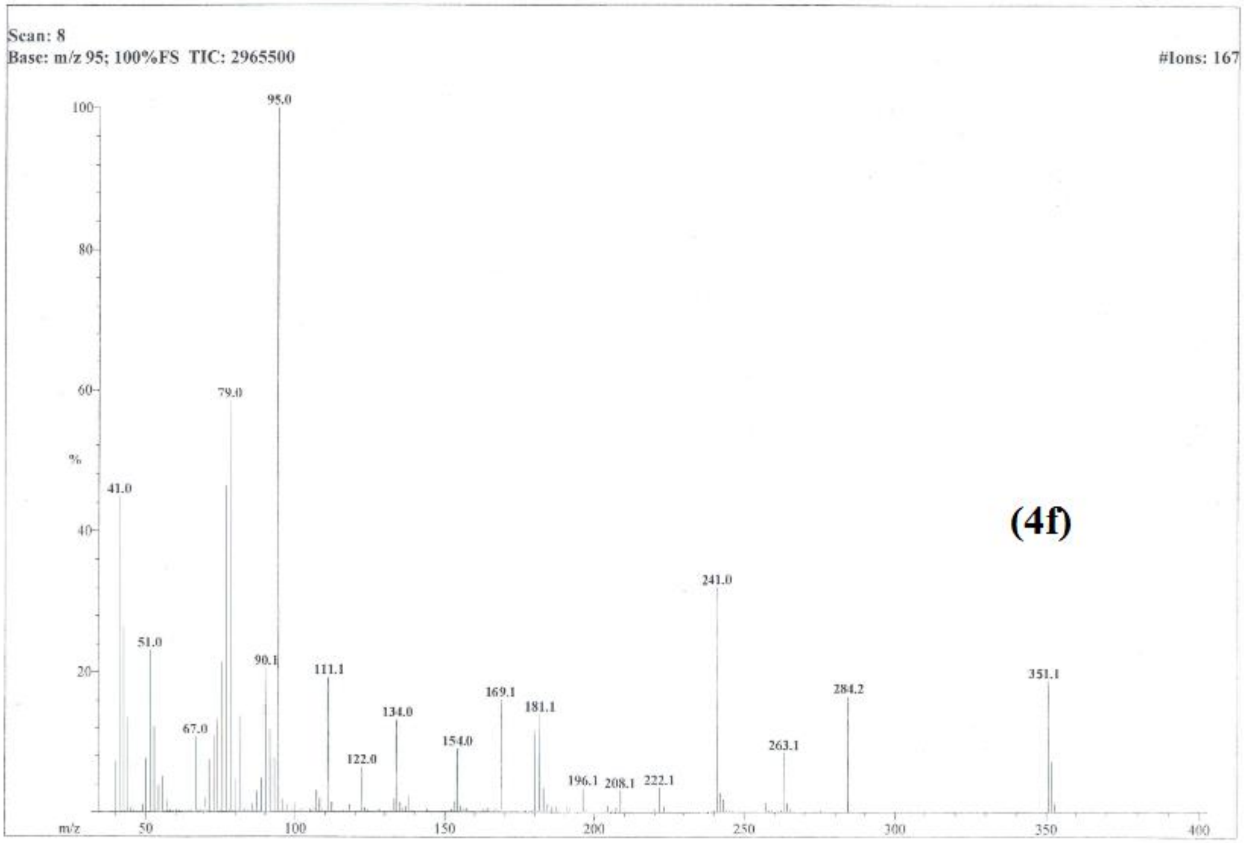

Strain: ***E.coli*** Strain***: S.aureus***

Front ***Back*** ***Front*** ***Back***

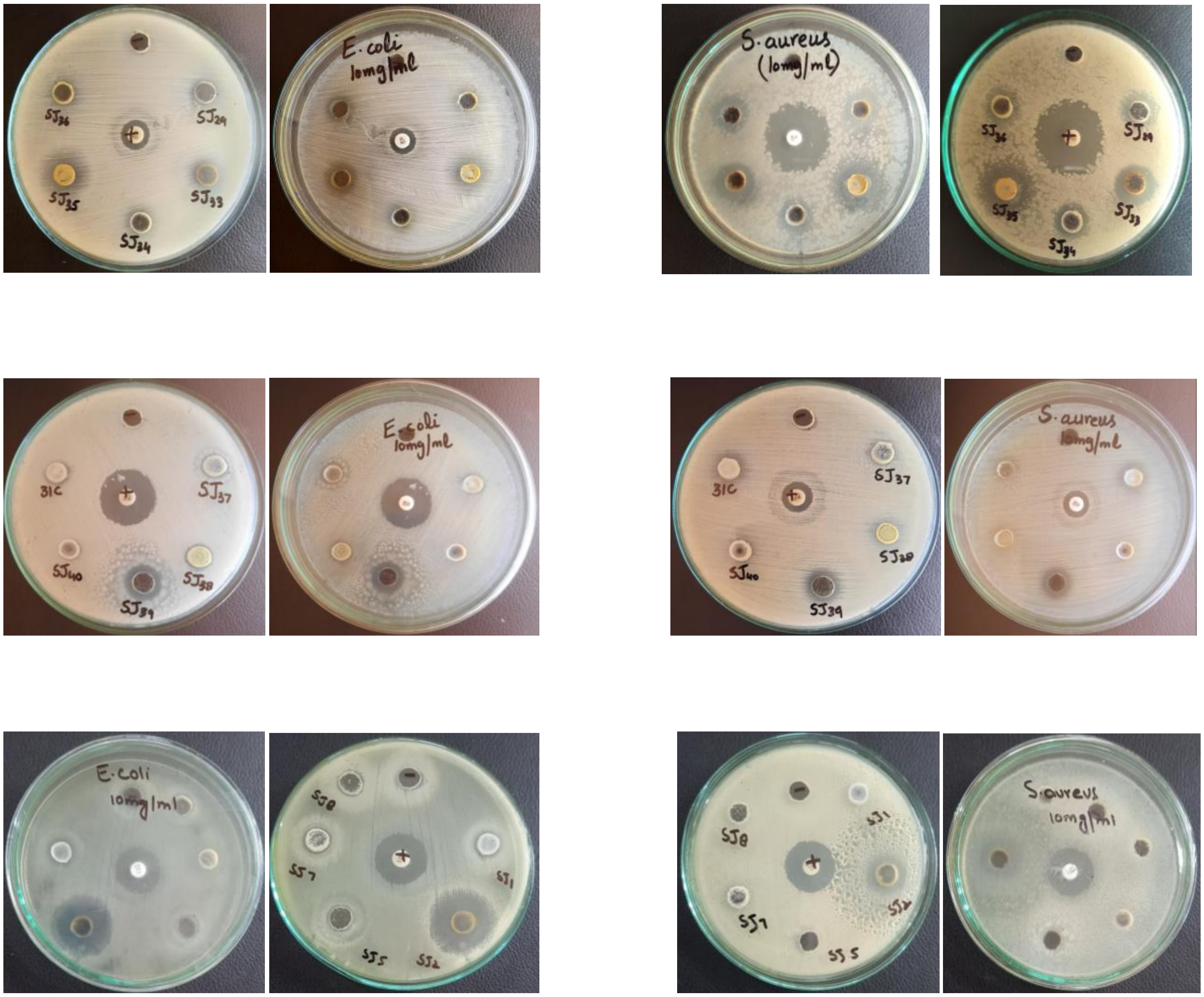

Note=S-J-36 (4a), S-J-37 (4b), S-J-38 (4c), S-J-34 (4d), S-J-8 (4e), S-J-35 (4f)

Figure S1. Antibacterial activity of the synthesized compounds (4a-4f) by well diffusion method.

Strain: *F. bracchygibossum*   Strain***: A. niger***

Front   ***Back***   ***Front***   ***Back***

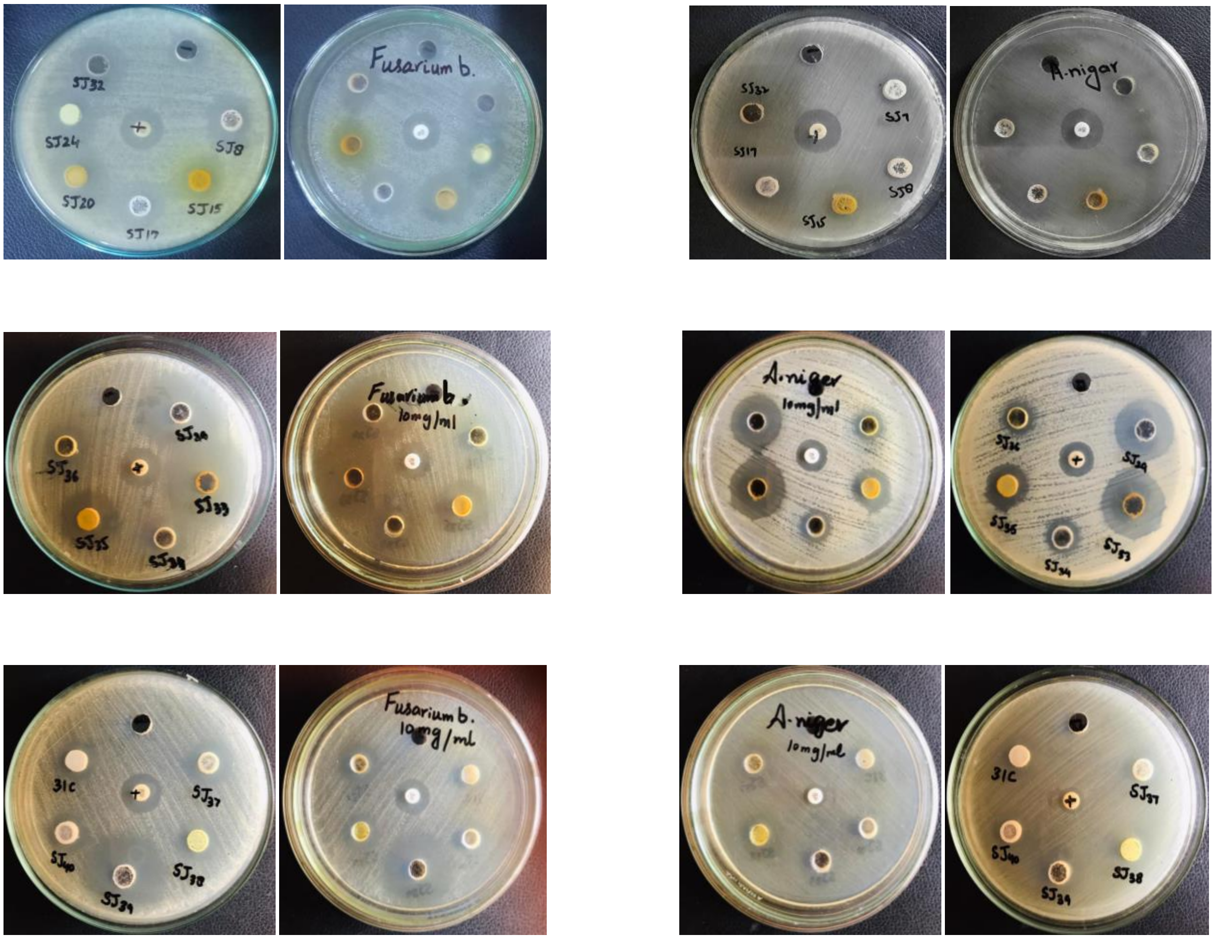

Note=S-J-36 (4a), S-J-37 (4b), S-J-38 (4c), S-J-34 (4d), S-J-8 (4e), S-J-35 (4f)

Figure S2. Antifungal activity of the synthesized compounds (4a-4f) by well diffusion method.

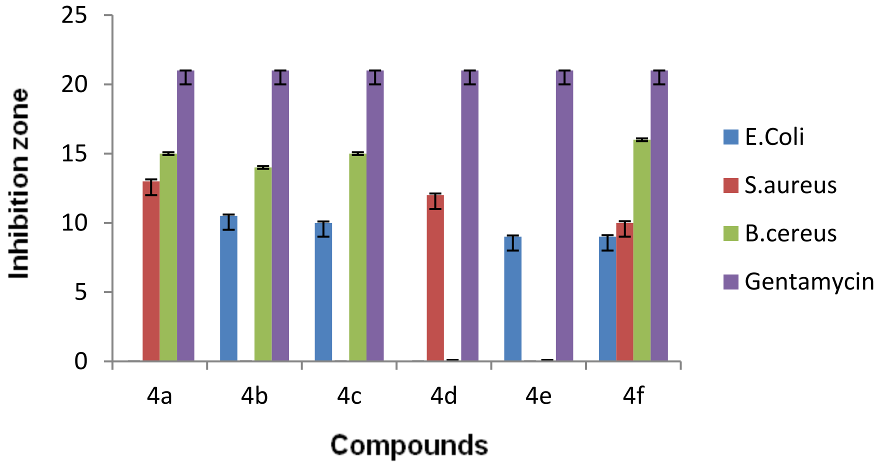

Figure S3: Antibacterial activity of the synthesized compounds (4a-4f) by well diffusion method.

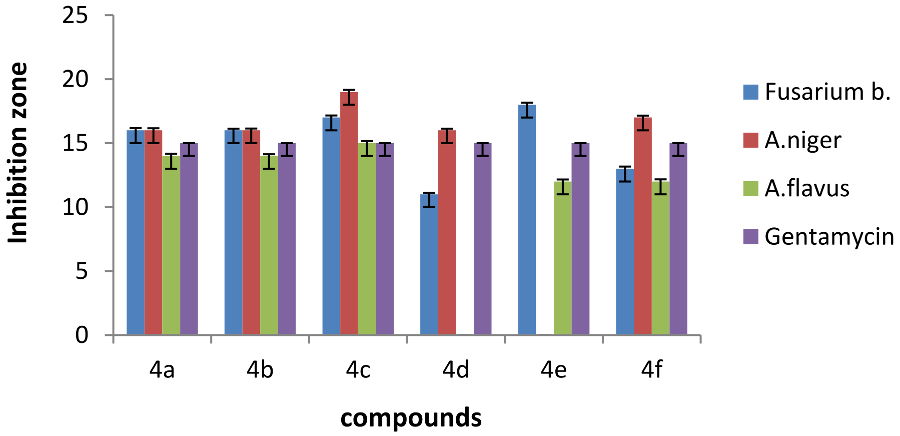

Figure S4: Antifungal activity of the synthesized compounds (4a-4h) by well diffusion method.
